# Ferroelectrics Hybrids: Harnessing Multifunctionality of 2D Semiconductors in the Post‐Moore Era

**DOI:** 10.1002/adma.202517269

**Published:** 2025-11-13

**Authors:** Haixin Qiu, Xiaoshi Qian, Dahong Qian, Paolo Samorì

**Affiliations:** ^1^ School of Biomedical Engineering Shanghai Jiao Tong University Shanghai 200240 China; ^2^ Université de Strasbourg CNRS, ISIS 8 allée Gaspard Monge Strasbourg 67000 France; ^3^ State Key Laboratory of Mechanical System and Vibration Interdisciplinary Research Center Institute of Refrigeration and Cryogenics and MOE Key Laboratory for Power Machinery and Engineering School of Mechanical Engineering Shanghai Jiao Tong University Shanghai 200240 China

**Keywords:** 2D semiconductors, ferroelectric materials, field‐effect transistors, molecular switches

## Abstract

The rise of big data in today's computing has highlighted the significant limitations of von Neumann architectures for data storage and processing. Concurrently, the downscaling of silicon‐based transistors while retaining low power efficiency and high system reliability has become increasingly challenging. By adopting a post‐Moore approach, this Review proposes the use of hybrid systems comprising ferroelectric materials, 2D semiconductors, and functional molecular switches to respond to current demands for simultaneous high integration density and multifunctional performance. The representative applications of 2D ferroelectric field‐effect transistors (FeFETs) are reviewed and advances in shrinking ferroelectric domain walls at the (sub)nanometer scale are highlighted. The incorporation of molecular switches to enable multimodal device programmability is explored and the implementation of monolithic 3D (M3D) integration to boost chip‐level density and system functionality is discussed. Finally, a forward‐looking vision is presented for future transistors built upon novel ferroelectric platforms. Taken together, this triple‐hybrid paradigm offers a compelling path to transcend Moore's law, paving the way for next‐generation electronics with unprecedented functions and performance.

## Graphical abstract

In this Review, the state of art of ferroelectric hybrid systems—combining ferroelectrics, 2D semiconductors, and molecular switches is presented—as next‐generation platforms for high‐density, multifunctional electronics. By discussing 2D FeFET applications, nanoscale material downscaling, M3D integration, and emerging ferroelectrics, it highlights strategies to overcome scaling bottlenecks and unlock device‐level multifunctionality.

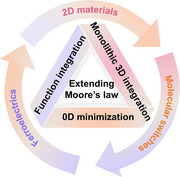

## 1 Introduction

**Figure 1:**
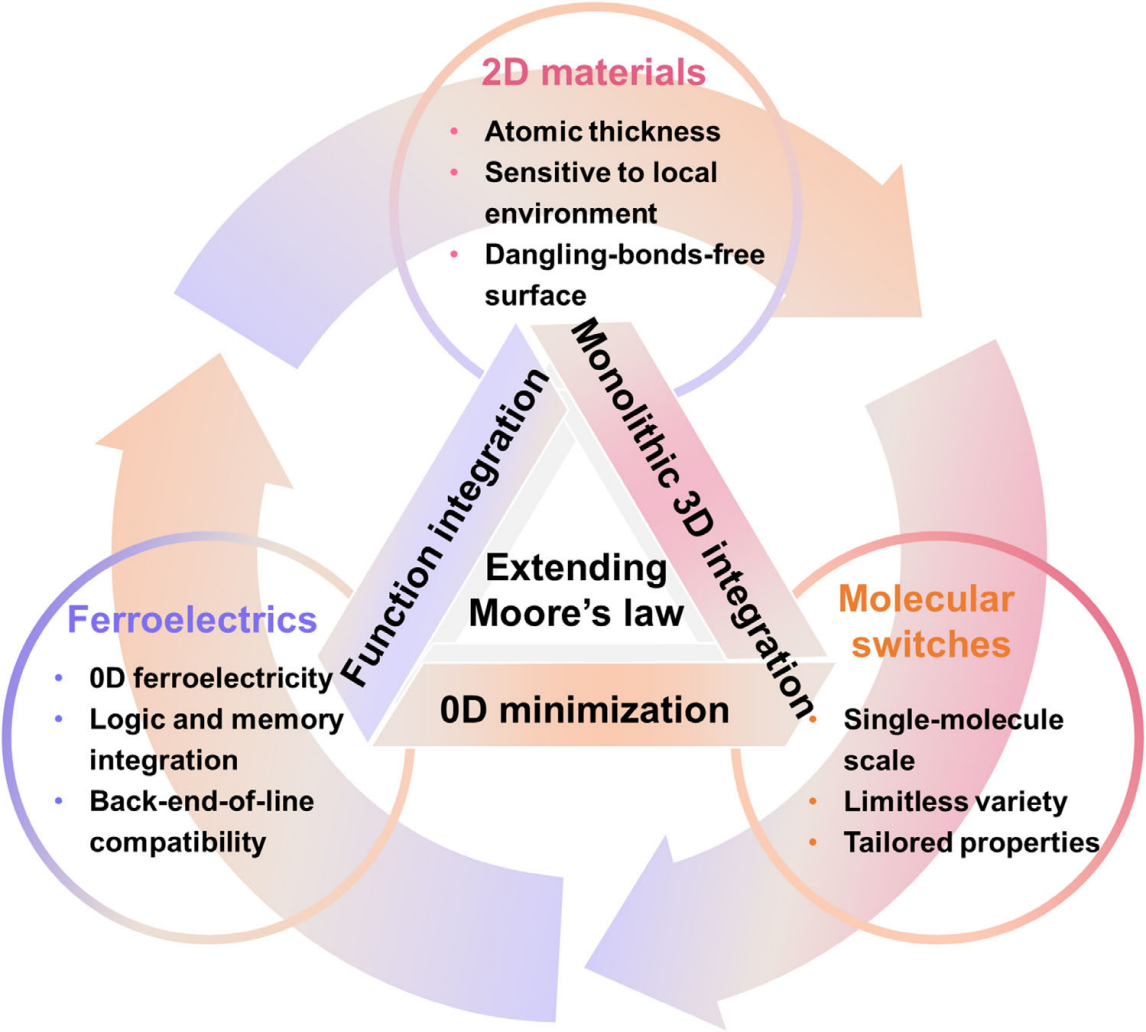
A hybrid platform comprising ferroelectric materials, 2D semiconductors, and functional molecular switches to address post‐Moore demands for simultaneous high integration density and multifunctional performance.

In the current era marked by the convergence of big data and artificial intelligence (AI), the once revolutionary von Neumann architecture has revealed its inadequacies in addressing the contemporary requirements for efficient data storage and processing. Concurrently, as the downscaling of silicon‐based transistors approaches its limits, challenges in power efficiency and system reliability are becoming increasingly pronounced.^[^ ^1^ ^]^ In the post‐Moore era, reconciling the divergences between conventional computational methodologies and nascent technological innovations demands a multi‐modal strategy.^[^ ^2^, ^3^ ^]^ Drawing inspiration from both More Moore and More than Moore technologies—which prioritize scaling and functional diversification, respectively—we propose a hybrid platform that integrates ferroelectric materials, 2D semiconductors, and functional molecular switches for the design of intelligent electronics (**Figure** **1**). As each component intrinsically supports both efficient down‐scaling and multifunctionality, their integration offers a promising route toward the effective combination of logic and memory at the (sub)nanometer scale, diversifying device operations, ultimately enabling monolithic 3D (M3D) integration for enhanced chip‐level density.

Traditional silicon‐based semiconductor technology suffers from inherent limitations in miniaturization due to their 3D crystal structure. In contrast, ferroelectric materials retain switchable polarization down to the 0‐D limit, enabling non‐volatile functionality at the atomic scale.^[^ ^4^ ^]^ Simultaneously, 2D semiconductors, with their in‐plane covalent bonding and atomic thickness, facilitates feature fabrication down to atomic thickness.^[^ ^5^ ^]^ Additionally, functional molecular switches offer ultimate size advantages—down to sub‐nanometer dimensions—along with structurally encoded properties that can be modulated at the molecular level.^[^ ^6^ ^]^ The integration of these three material classes offers a powerful strategy for constructing ultrascaled transistors, providing a viable pathway to extend More Moore scaling.

In addition to miniaturization, the integration of multifunctionality and multiresponsiveness in electronic devices represents another critical frontier in the post‐Moore era. Ferroelectric materials, with intrinsic binary polarization states, inherently enable the integration of logic and memory functions within a single device, aligning them with cutting‐edge applications in neuromorphic computing, AI, and machine learning. Moreover, owing to the strong coupling among polarization, lattice, and charge degrees of freedom, ferroelectric polarization can be modulated not only by electric field but also by a variety of other physical stimuli such as optical, mechanical, thermal, and magnetic fields, significantly enhancing their functional versatility. 2D semiconductors, with their high surface‐to‐volume ratios and atomic‐level sensitivity, serve as ideal platforms for interfacing with external signals, enabling responsive electronic, optoelectronic, and sensing functionalities. When integrated with ferroelectrics, they unlock novel operating principles and enhanced performance. Molecular switches further enrich their functions by transducing diverse physical, chemical, or biological signals into electrical outputs via nanoscale conformational or electronic changes. The synergistic integration of ferroelectric materials, 2D semiconductors and functional molecular switches thus enables the creation of multifunctional devices that surpass the limitations of traditional electric‐field‐driven architectures. Although the library of experimentally mature ferroelectric and 2D semiconductors remains limited, the vast chemical diversity of molecular switches offers a nearly limitless design space. Combined with their low‐temperature processability and compatibility with back‐end‐of‐line (BEOL) fabrication, such hybrid systems are highly suited for M3D integration—advancing both device density and functional complexity in pursuit of More than Moore technologies.^[^ ^7^ ^]^

As ferroelectric hybrid systems emerge as a promising platform for next‐generation electronics, their development sits at the intersection of unprecedented opportunities and critical challenges. This Review provides a systematic overview of recent progress in ferroelectric hybrid systems for integrated electronics, tracing their evolution from extreme dimensional scaling to functional diversification and ultimately to M3D architectures. We highlight the critical technological and material obstacles that emerge at sub‐10‐nanometer nodes, and persistent barriers to functional integration that constrain device performance. Particular attention is given to strategies developed to overcome these limitations, as well as to prospective approaches that may unlock further scalability and versatility. Moreover, a growing library of ferroelectric materials with exceptional properties offers exciting prospects for future device platforms—though their integration into practical systems remains largely underexplored. By elucidating their fundamental characteristics and system‐level implications, this Review aims to bridge the gap between materials advances and integrated circuit design, offering a roadmap toward future innovations in computation, sensing, and intelligent electronics in the post‐Moore era.

## 2 2D Ferroelectric Field‐Effect Transistors (FeFETs) for Next‐Generation Digital Electronics

FeFETs are emerging as key enablers for next‐generation low‐power, high‐performance, and multifunctional electronic systems. By integrating ferroelectric materials as the gate dielectric to modulate the conductivity of the semiconductor channel, FeFETs inherently combine non‐volatile data retention, steep subthreshold switching, and logic‐in‐memory functionality, representing critical attributes for addressing the scaling bottlenecks and energy‐efficiency constraints of conventional CMOS technologies.

**Table** **1** summarizes the key performance parameters of five representative FeFET classes—MOS FeFETs, 2D FeFETs, 2D van der Waals (vdW) FeFETs, FeSFETs, and vdW FeSFETs—categorized according to their gate‐stack configuration and channel dimensionality. Traditional MOS FeFETs, typically based on bulk ferroelectrics in conjunction with silicon‐based channels, have demonstrated reliable memory operation.^[^ ^8^, ^9^, ^10^, ^11^, ^12^ ^]^ However, their further integration with modern semiconductor platforms faces inherent challenges, including lattice mismatch, interfacial instability, and suboptimal electrostatic control, which are exacerbated at reduced dimensions.^[^ ^13^, ^14^, ^15^ ^]^ These limitations not only degrade retention and endurance but also introduce undesirable hysteresis and charge trapping, particularly when the remanent ferroelectric polarization exceeds the charge‐handling capacity of the silicon channel. In contrast, 2D FeFETs that couple ferroelectric materials with atomically thin semiconductors offer a compelling pathway to overcome these challenges. Owing to their reduced dimensionality, 2D semiconductors provide enhanced electrostatic control, minimized interface trap densities, and efficient electrostatic coupling to the ferroelectric polarization field. These features enable effective modulation of the channel carrier concentration in response to remanent polarization P_r_, thereby mitigating depolarization effects. Moreover, the excellent interface quality and flexibility of 2D layered assembly allow for scalable and defect‐tolerant device fabrication. These synergistic advantages position 2D FeFETs as promising candidates for next‐generation memory and logic‐in‐memory technologies. A further evolution is represented by vdW FeFETs, in which both the ferroelectric and semiconductor components are formed from van der Waals materials. Clean, dangling‐bond‐free interfaces between vdW layers effectively suppress defect formation and interfacial trap states, enhancing stability and scalability. FeSFETs, in which the active channel itself is a ferroelectric semiconductor, provide additional design flexibility. The presence of intrinsic mobile carriers generates internal electric fields that screen depolarization and reduce leakage current, thereby improving retention and switching uniformity. Finally, vdW FeSFETs, combining vdW ferroelectric dielectrics with ferroelectric semiconducting channels, establish a fully 2D platform that unites superior scalability, reduced defect density, and mechanical flexibility, making them particularly attractive for ultrathin, flexible electronic systems.

**Table 1:** Comparison of the key parameters for representative FeFETs based on metal‐oxide ‐semiconductor, 2D semiconductors, ferroelectric semiconductors, and vdW ferroelectric stacks.

Classification	Gate dielectric	Channel	Memory window vs sweep voltage	ON/OFF ratio	SS [mVdec^−1^]	Retention time	Endurance	Refs.
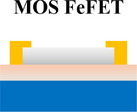	HZO	ZnO	1.9 vs 6	10^6^	133	10^4^ s extrapolated to 10 years	10^8^	[8]
	HZO	ITO	2.8 vs 10	10^8^	33	10^4^ s extrapolated to 10 years	10^7^	[9]
	HZO	Si	0.5 vs 3.5	10^7^	/	10^5^ s extrapolated to 10 years	10^7^	[10]
	HZO	In_2_O_3_	6 vs 12	10^7^	123	10^4^ s extrapolated to 10 years	10^7^	[11]
	HZO	In_2_O_3_	2.2 vs 8	10^7^	/	10^4^ s extrapolated 10 years	10^8^	[12]
	HZO	IGZO	1 vs 6	10^5^	113	/	10^4^	[13]
	TaN/HZO	Si	0.7 vs 9.6	10^4^	/	10^4^ s extrapolated to 10 years	10^3^	[14]
	PZT	IGZO	3 vs 10	10^6^	1250	10^3^ s	/	[15]
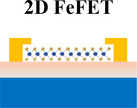	P(VDF‐TrFE)	MoS_2_	14 vs 20	10^3^	300	10^3^ s	/	[17]
	PZT	MoS_2_	/	10^6^	9.7	/	/	[18]
	BTO	MoS_2_	44 vs 100	10^7^	/	/	/	[19]
	P(VDF‐TrFE)	MoSe_2_	30 vs 60	10^5^	/	10^3^ s	10^4^	[20]
	PZT	WSe_2_	3 vs 8	10^5^	/	10 d	400	[21]
	P(VDF‐TrFE)	BP	15 vs 60	10^3^	/	3500 s	/	[22]
	AlScN	MoS_2_	8 vs 20	10^7^	750	10^5^ s extrapolated to 10 years	10^4^	[25]
	Al_2_O_3_/HZO	MoS_2_	0.012 vs 1.5	10^7^	5.6	/	/	[77]
	Al_2_O_3_/HZO	MoS_2_	0.48 vs 2	10^7^	51.2	10^4^ s	10^3^	[78]
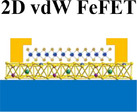	CIPS	MoS_2_	2 vs 6	10^7^	22	10^3^ s	10^3^	[35]
	CIPS	WSe_2_	6 vs 12	10^6^	/	10^3^ s	320	[36]
	CIPS/graphene/hBN	MoS_2_	3.8 vs 8	10^7^	17	10 h extrapolated to 10 years	10^4^	[37]
	CIPS/hBN	ReS_2_	2.8 vs 10	10^5^	/	10^3^ s	/	[38]
	CIPS/hBN	SnS_2_	18.4 vs 50	10^5^	/	10^4^ s	350	[39]
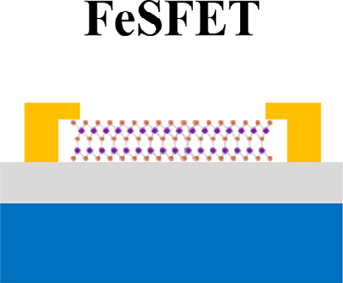	Al_2_O_3_	r‐stacked MoS_2_	4.6 vs 20	10^6^		10 years	10^4^	[43]
	Al_2_O_3_	Bi_2_O_2_Se	4.7 vs 10	10^4^	/	/	/	[49]
	Al_2_O_3_	α‐In_2_Se_3_	15 vs 20	10^5^	/	/	250	[51]
	Al_2_O_3_	α‐In_2_Se_3_	0.2 vs 3	10^5^	/	10^5^ s extrapolated to 10 years	10^6^	[100]
	HfO_2_	α‐In_2_Se_3_	4 vs 10	10^5^	/	/	10^5^	[101]
	HfO_2_	α‐In_2_Se_3_	4 vs 6	10^8^	500	/	/	[40]
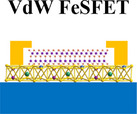	hBN	r‐stacked MoS_2_	7 vs 20	10^6^	/	10^4^ s extrapolated to years	10^4^	[50]
	hBN	α‐In_2_Se_3_	6 vs 16	10^5^	/	500 s	500	[52]
	CIPS/hBN	α‐In_2_Se_3_	14 vs 20	10^6^	/	10^4^ s	10^4^	[53]
	CIPS/hBN	SnS	70 vs 120	10^5^		10^4^ s	10^4^	[54]
	CIPS/hBN	InSe	4.6 vs 10	10^4^	/	10^3^ s extrapolated to 10 years	10^3^	[41]

This section systematically reviews the recent progress of 2D FeFETs, with particular emphasis on their applications in non‐volatile memories, neuromorphic computing, reconfigurable logic circuits, negative capacitance field‐effect transistors (NC‐FETs), and multifunctional FeFETs. We highlight that the coupling between ferroelectric polarization and the electronic/structural properties of 2D materials enables novel device functionalities while simultaneously addressing persistent integration challenges. Taken together, these advances establish 2D/ferroelectric hybrid FeFETs as a versatile platform that not only transcends Moore‐era limitations but also opens a pathway toward programmable, compact, and energy‐efficient electronic systems of the future.

### 2.1 Non‐Volatile Memories

In non‐volatile memory architectures, FeFETs uniquely leverage ferroelectric polarization switching to encode and retain information within the transistor channel. By employing ferroelectric materials as gate dielectrics, the device conductance can be reversibly modulated between bistable high‐ and low‐resistance states through purely electrical gating, thereby eliminating standby power consumption and enabling logic‐in‐memory operation.^[^ ^16^ ^]^ This charge‐based switching mechanism offers a compact and energy‐efficient alternative to conventional floating‐gate or charge‐trap memory devices.

**Figure 2:**
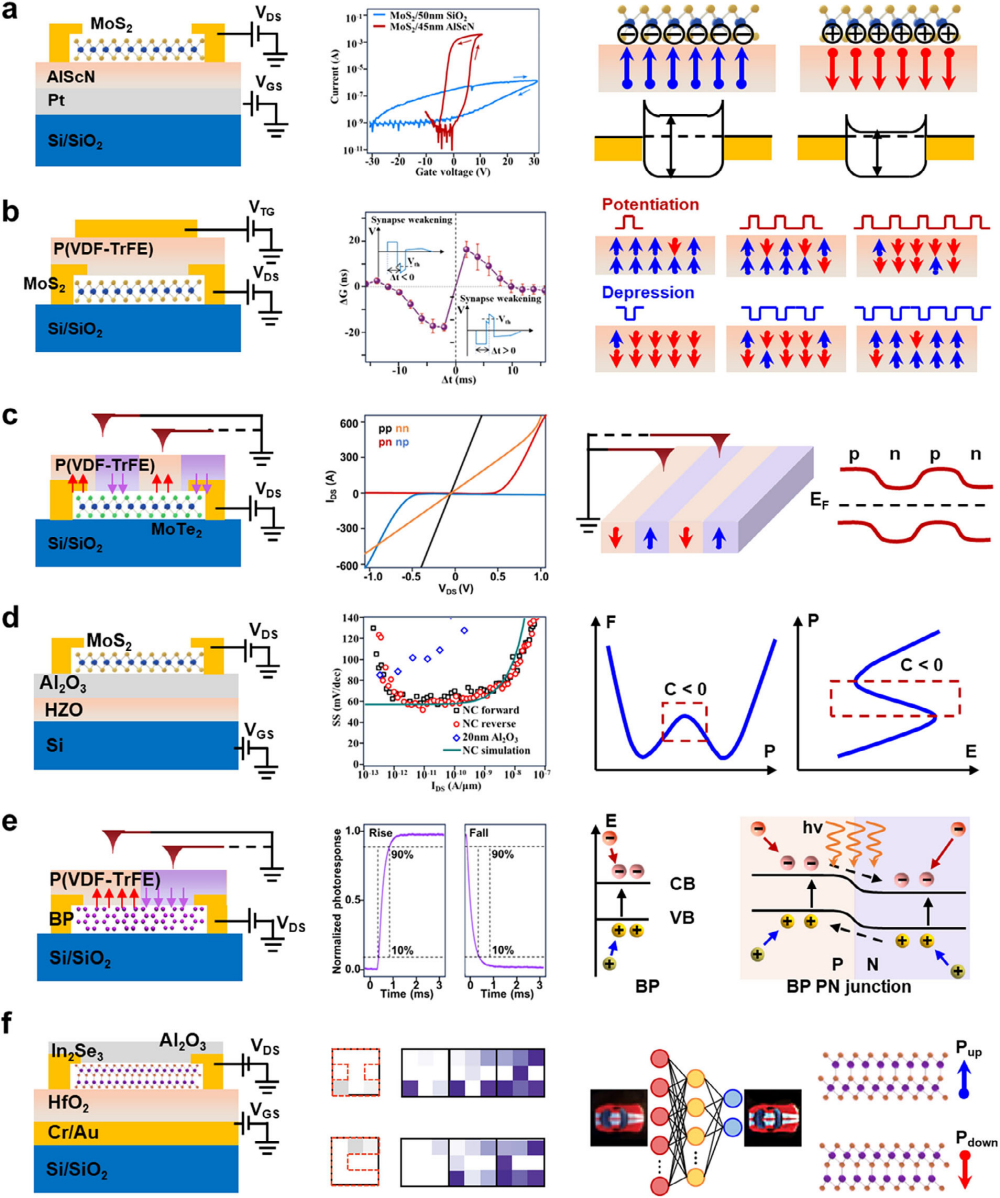
Representative 2D FeFETs for next‐generation digital electronics, device archtection, functionalities and operation mechanisms. a) Schematic of a MoS_2_/AlScN FeFET, and its semilogarithmic‐scale transfer characteristics. Reproduced with permission.^[^ ^25^ ^]^ Copyright 2023, Springer Nature. b) Schematic of a MoS_2_/P(VDF‐TrFE) FeFET synapse, and its STDP behavior characterization. Reproduced with permission.^[^ ^48^ ^]^ Copyright 2019, Wiley. c) Schematic of a MoTe_2_/P(VDF‐TrFE) reconfigurable FeFET, and its output curves for the p‐p, p‐n, n‐p and n‐n homojunctions. Reproduced with permission.^[^ ^72^ ^]^ Copyright 2020, Springer Nature. d) Schematic of a MoS_2_/Al_2_O_3_/HZO NC‐FET, and its SS versus I_DS_ characteristics. Reproduced with permission.^[^ ^77^ ^]^ Copyright 2018, Springer Nature. e) Schematic of a BP/P(VDF‐TrFE) FeFET photodetector, and its rise and decay time. Reproduced with permission.^[^ ^95^ ^]^ Copyright 2022, Springer Nature. f) Schematic of a In_2_Se_3_ ferroelectric‐semiconductor‐transistor, and its evolutions of the electrical output images after being illuminated by optical input images of letter “I” and “C” (above), a back‐end neuromorphic feature extraction and object recognition process (below). Reproduced with permission.^[^ ^101^ ^]^ Copyright 2023, Wiley.

The integration of 2D semiconductors as channel materials further amplifies the advantages of FeFETs in memory applications.^[^ ^17^, ^18^, ^19^ ^]^ Their atomically thin geometry ensures strong electrostatic coupling and minimizes interface trap densities, both of which are critical for efficient polarization‐charge interaction.^[^ ^20^, ^21^, ^22^ ^]^ Unlike traditional silicon‐based metal‐oxide‐semiconductor field‐effect transistors (MOSFETs) with SiO_2_ gate dielectrics that sustain only ≈10^13^ cm^−2^ of inversion charge density,^[^ ^23^ ^]^ 2D semiconductors can support much higher sheet carrier densities ranging from 2 × 10^13^ to 1 × 10^15^ cm^−2^, providing an excellent match to the P_r_ charge densities induced by ferroelectric materials such as HZO (≈10^14^ cm^−2^).^[^ ^24^ ^]^ This compatibility mitigates charge mismatch and suppresses associated issues such as interface trapping, depolarization, and retention degradation, thereby enabling more stable and efficient memory operation. A representative demonstration by Jariwala et al. integrated ferroelectric AlScN thin films with monolayer MoS_2_ to fabricate scalable 2D FeFETs. The resulting devices exhibited a memory window exceeding 7.8 V, ON/OFF current ratios above 10^7^, excellent endurance and reliable non‐volatility (**Figure** **2**a).^[^ ^25^ ^]^

Building on these promising demonstrations, the integration of ferroelectric memories into next‐generation electronic platforms demands simultaneous optimization of multiple parameters, including sub‐1 V operating voltage for energy efficiency, high P_r_ for data density, and robust fatigue endurance exceeding 10^15^ cycles to guarantee a decade‐long reliability. Achieving these metrics concurrently remains a formidable challenge, largely due to the intrinsically high *E*_c_ of most ferroelectrics, which often approaches 60% to 80% of the material's breakdown threshold.^[^ ^26^ ^]^ This narrow design margin accelerates polarization fatigue, as repeated switching generates and accumulates defects—oxygen vacancies in oxides or trap states in layered materials—that destabilize polarization and eventually trigger hard breakdown.^[^ ^27^ ^]^

One effective approach to reduce *E*_c_ involves the doping‐induced stabilization of the intermediate nonpolar phase, which lowers the energy barrier between bistable polarization states. In hafnia‐based ferroelectrics, chemical doping can stabilize the tetragonal (t‐) phase that lies between the two orthorhombic polar states. For example, lanthanum doping has been shown to increase the formation energy of oxygen vacancies, thereby suppressing defect generation during repeated switching cycles. This not only reduces *E*_c_ by nearly half but also enhances endurance, with stable polarization switching demonstrated up to 10^10^ cycles.^[^ ^28^, ^29^, ^30^, ^31^ ^]^ However, excessive stabilization of the t‐phase diminishes remnant polarization and compromises retention, illustrating the trade‐offs involved in optimizing reliability‐related parameters.

Retention degradation in FeFETs arises from multiple factors, including depolarization fields, charge trapping, leakage current, and imprint effects.^[^ ^32^ ^]^ Owing to the ultrathin ferroelectric films (<15 nm) required for memory applications, the electrode‐ferroelectric interface plays a particularly critical role.^[^ ^33^ ^]^ However, fabrication‐induced interfacial oxides, stress accumulation, and chemical diffusion can significantly disturb the structural and compositional integrity of the ferroelectric stack.^[^ ^33^ ^]^ To mitigate these effects, tuning the electrode work function and minimizing interfacial reactivity have proven effective in enhancing retention and reliability. For instance, the use of inert electrodes effectively preserves polarization stability by limiting undesired interfacial reactions.^[^ ^33^ ^]^ Similarly, interface passivation methods—such as NH_3_ plasma treatment in hafnia‐based systems—reduce oxygen vacancy concentration near the interface, improving charge retention and endurance.^[^ ^34^ ^]^

To further address interface‐related retention degradation, van der Waals (vdW) engineering has emerged as an alternative strategy. By assembling vdW ferroelectrics with a broad range of 2D semiconductors and insulators via clean, dangling‐bond‐free interfaces, this method minimizes defect formation and suppresses interfacial trap states. Notable examples include CuInP_2_S_6_ (CIPS) integrated 2D vdW FeFETs, which have demonstrated exceptional data retention over ten years.^[^ ^35^, ^36^, ^37^, ^38^, ^39^ ^]^ Going a step further, ferroelectric semiconductor FETs (FeSFETs)—which leverage 2D ferroelectric semiconductors as active channel layers—offer further design advantages.^[^ ^40^, ^41^ ^]^ The presence of intrinsic mobile carriers in these semiconductors generates internal electric fields that effectively screen depolarization fields, thereby mitigating charge trapping and reducing leakage current, collectively boosting retention and switching stability.^[^ ^42^ ^]^ In this regard, atomically thin 2D sliding ferroelectrics align with the requirements. Notably, FeSFETs with polarity‐switchable epitaxial rhombohedral‐stacked MoS_2_ as ferroelectric channel have exhibited robust non‐volatile memory behavior at thickness of only two atomic layers.^[^ ^43^ ^]^

Beyond FeFETs and FESFETs utilizing ferroelectric gates or ferroelectric semiconducting channels, a recently developed vertical tunneling FeFET introduces a fundamentally different device architecture based on a vdW MoS_2_/h‐BN/metal tunnel junction serving as the channel.^[^ ^44^ ^]^ In this configuration, ferroelectric polarization modulates the Fermi level of MoS_2_, thereby controls the quantum tunneling current across the h‐BN barrier. The design effectively combines the non‐volatile polarization control of ferroelectrics with the precise quantum tunneling modulation of 2D junctions. The resulting device exhibits ON/OFF ratios up to 10^9^ and ultralow energy consumption of only 0.16 fJ per switching event, highlighting a promising avenue for the design flexibility of ferroelectric memory technologies. Furthermore, continued efforts on novel device geometries, such as elliptic junctionless Gate‐All‐Around FeFETs, aim to deepen the theoretical framework governing electrostatics and scaling behavior in ferroelectric transistors, laying the groundwork for next‐generation ferroelectric logic and memory architectures.^[^ ^45^ ^]^

In addition to material and interface considerations, thickness scaling of ferroelectric films has emerged as another critical route to enhance performance. In hafnia‐based systems, reducing film thickness below 10 nm has revealed a near‐linear relationship between film thickness and coercive voltage, enabling low‐voltage operation. Furthermore, ultrathin films exhibit improved breakdown resistance due to reduced carrier injection energy from electrodes and minimized structural damage.^[^ ^46^ ^]^ For instance, a 4 nm‐thick HZO capacitor has demonstrated an operating voltage as low as 1.2 V and endurance up to 10^12^ cycles at 100 kHz, with potential to exceed 10^14^ cycles through further scaling of device area and operation frequency.^[^ ^46^ ^]^

Collectively, these synergistic strategies—doping for phase stabilization, interface engineering for retention, vdW integration for defect suppression, and thickness scaling for voltage optimization—mark significant progress toward industrial‐grade FeFETs. With these advances, FeFET technology is rapidly transitioning from academic proof‐of‐concept to scalable, reliable platforms for next‐generation non‐volatile memory.

### 2.2 Neuromorphic Computing

The application scope of FeFETs has expanded considerably beyond traditional non‐volatile memories. By leveraging the hysteretic and history‐dependent characteristics of ferroelectric polarization, FeFETs intrinsically emulate the adaptive behavior of biological synapses.^[^ ^47^ ^]^ In particular, three‐terminal FeFET architectures—where the gate and drain terminals correspond to pre‐ and postsynaptic neurons, respectively—enable decoupled learning and signal transmission within a single device. The channel conductance, modulated by the P_r_ of the ferroelectric gate, provides an analog encoding of synaptic weight. This configuration facilitates hardware‐level implementation of key synaptic functionalities, including long‐term potentiation (LTP), long‐term depression (LTD), and spike‐timing‐dependent plasticity (STDP), while maintaining ultralow energy consumption.

The integration of 2D semiconductors into FeFET structures introduces additional degrees of freedom for synaptic modulation. Their atomically thin structure allows for precise electrostatic doping and supports analog multilevel conductance tuning. For example, FeFETs based on MoS_2_/P(VDF‐TrFE) heterostructures exhibit over 1000 stable intermediate states through partial polarization switching, faithfully mimicking the stimulus‐dependent plasticity in biological synapses (Figure 2b).^[^ ^48^ ^]^ Specifically, downward ferroelectric polarization accumulates carriers in MoS_2_ and increases conductance of the device, while upward polarization depletes carriers, leading to lower conductance. This polarity‐dependent modulation generates wide conductance hysteresis and facilitates finely tunable, amplitude‐ and duration‐dependent weight updates. Furthermore, such FeFET synapses support STDP, a key Hebbian learning rule where the relative timing (Δt) between pre‐ and postsynaptic spikes determines synaptic weight updates. Positive Δt leads to LTP, while negative Δt induces LTD, with the extent of modification decaying as |Δt| increases. This biologically faithful temporal response is combined with excellent energy efficiency (below 1 fJ per synaptic operation) and extended operational lifespans (≈10 years working as a biological brain work frequency of 10 Hz). These characteristics strongly position 2D FeFET‐based synapses as viable candidates for brain‐inspired neuromorphic computing.

In addition to conventional FeFET architectures, FeSFETs based on intrinsically ferroelectric 2D materials such as α‐In_2_Se_3_, Bi_2_O_2_Se offer a simplified and highly integrated approach for neuromorphic device design.^[^ ^49^ ^]^ Unlike FeFETs that require a separate ferroelectric gate stack, FeSFETs leverage the coexisting ferroelectricity and semiconducting properties within a single material phase. This enables non‐volatile conductance modulation via polarization switching, while multiple intermediate conductance states can be programmed by manipulating the out‐of‐plane polarization direction. Such tunability allows the emulation of key synaptic behaviors such as LTP and LTD. Compared to charge‐trapping‐based synaptic devices, α‐In_2_Se_3_‐based vdW FeSFETs exhibit faster learning, greater controllability, and structural simplicity, enhancing their scalability for neuromorphic hardware.^[^ ^50^, ^51^, ^52^ ^]^ Additionally, an abnormal resistive switching effect observed under in‐plane polarization operation provides further degrees of freedom for state tuning. Together, these findings emphasize the promise of 2D ferroelectric semiconductors as a promising class of neuromorphic building blocks.^[^ ^53^, ^54^ ^]^

Despite their promise for energy‐efficient neuromorphic computing, ferroelectric‐based artificial synapses often exhibit nonlinear and asymmetric weight updates under identical voltage pulses. This behavior mainly arises from the intrinsic nonlinear switching characteristics of ferroelectrics, where initial domain nucleation leads to a rapid conductance change followed by a slower evolution as domains expand.^[^ ^55^ ^]^ This imbalance between LTP and LTD deteriorates learning accuracy and network stability in STDP‐based models.^[^ ^56^ ^]^ To overcome these limitations, efforts have focused on optimizing pulse schemes, such as pulse‐width or amplitude modulation, to linearize conductance changes.^[^ ^57^ ^]^ Hardware‐software co‐optimization through adaptive learning algorithms has also been explored to compensate for intrinsic device nonlinearity.^[^ ^58^ ^]^ Furthermore, coupling ion migration with ferroelectric domain dynamics has been shown effective. Under an external electric field, oxygen vacancy migration can tune the interfacial potential drop and slow domain nucleation, yielding smoother and symmetric weight evolution.^[^ ^59^ ^]^ A representative Au/Cr/BaTiO_3_/Nb:SrTiO_3_ ferroelectric tunneling junction achieved highly linear and symmetric conductance changes by combining ferroelectric polarization switching with controlled oxygen‐vacancy migration. When implemented in neural network simulations, this device achieved a supervised learning accuracy of 96.7% and exhibited stable, noise‐resilient unsupervised learning behavior. Collectively, these advances pave the way toward more reliable and energy‐efficient neuromorphic systems.^[^ ^59^ ^]^

As neuromorphic devices progress from emulating individual synaptic behaviors toward system‐level implementation, the requirements imposed by AI workloads become increasingly critical. Since AI applications increasingly demand real‐time adaptability, on‐device learning, and energy‐efficient autonomy, making the integration of both inference and training within the same hardware indispensable. However, this remains a formidable challenge due to the inherently divergent memory requirements of the two processes: inference primarily relies on pre‐stored weights and demands long‐term retention with moderate precision, while training involves frequent weight updates, requiring high endurance, fast switching, and low energy consumption.^[^ ^60^, ^61^ ^]^ This divergence has hindered the development of universal memory architectures capable of supporting both processes simultaneously, thereby limiting progress toward efficient, fully integrated AI hardware.

To overcome this bottleneck, 2D FeFET synapses offer unique advantages. Their ultrafast polarization switching, low operating voltages, and analog conductance tunability enable in‐memory computing with reduced data‐transfer overhead between memory and logic. Unlike traditional volatile processors, their non‐volatile and reconfigurable conductance states allow both training and inference to be executed locally within the same memory array. When integrated into deep neural networks, they offer significant improvements in speed and energy efficiency for data‐intensive tasks such as image and speech recognition.^[^ ^62^ ^]^ In the domain of convolutional neural networks, their ability to handle data swiftly and accurately aids in pattern and object detection methodologies.^[^ ^63^, ^64^ ^]^ Furthermore, within recurrent neural networks, they show immense promise for augmenting sequence analysis and predictive modelling, which are integral to complex tasks like language processing and time series analysis.^[^ ^65^ ^]^ Additionally, their application in spiking neural networks heralds the advent of more biologically realistic models of neural processing, opening new avenues in computational neuroscience and AI.^[^ ^66^ ^]^ Pushing beyond single‐device demonstrations, a novel duplex device structure has been exploited based on a FeFET coupled with a monolayer MoS_2_ channel to reconcile training and inference within one platform. By leveraging the tunable double‐well energy landscape of ferroelectrics, this design enables the simultaneous execution of both training and inference through edge computation.^[^ ^67^ ^]^ The duplex building block demonstrates excellent device‐level metrics, including endurance exceeding 10^13^ cycles, data retention over 10 years, ultrafast speed of 4.8 ns, and low energy consumption of 22.7 Fj bit^−1^ µm^−2^. Notably, when implemented in a multilayer neural network using two‐FETs‐one‐duplex FeFET cells, it achieves 99.86% accuracy on a nonlinear localization task with in situ trained weights, marking a critical step toward adaptive, energy‐efficient, and fully integrated neuromorphic systems.

Looking forward, monolithic integration of such duplex FeFET cores with essential neuromorphic processing elements—such as activation, pooling, and routing—could facilitate fully in‐memory computation and end‐to‐end edge intelligence. This vision supports a broader shift toward autonomous, real‐time learning systems that minimize reliance on cloud resources, reduce latency, and enhance privacy. Such FeFET‐based neuromorphic hardware is thus well positioned to address the next‐generation AI challenges spanning from wearable devices to smart robotics and adaptive human‐machine interfaces.

### 2.3 Reconfigurable Logic Circuits

As conventional CMOS technology approaches scaling bottlenecks, in particular below the 10 nm node, reconfigurable FETs have emerged as a promising solution to reduce circuit complexity and enhance logic versatility. FeFETs stand out in this context because their switchable polarization enables non‐volatile reconfiguration of both logic polarity and device functionality. Unlike traditional transistors with fixed n‐ or p‐type conduction, FeFETs leverage ferroelectric polarization to modulate the band alignment at the ferroelectric/semiconductor interface, thereby dynamically switching the channel between electron‐ and hole‐dominated transport. This electrostatic reprogramming is non‐volatile and bidirectional, allowing a single transistor to perform multiple logic roles and minimizing both transistor count and energy consumption. A reconfigurable FeFET typically employs dual‐gate control, where the program gate defines the carrier type and the control gate toggles the transistor ON and OFF. This disruptive concept allows reversible transitions between n‐ and p‐type operating modes.^[^ ^68^ ^]^ As a result, circuit complexity can be substantially reduced for a given functionality. Circuit‐level evaluations have quantified ≈20% lower normalized delay, ≈32% smaller area, and ≈40% lower activity compared with CMOS reference of identical functionality, highlighting the performance and energy‐efficiency advantages of reconfigurable transistor architectures.^[^ ^69^ ^]^

The integration of 2D semiconductors such as WSe_2_ or MoTe_2_ further amplifies the potential of reconfigurable FeFETs, owing to their intrinsic ambipolar transport and efficient electrostatic gate controllability.^[^ ^70^, ^71^ ^]^ In 2D/ferroelectric heterostructures, the polarization field of the ferroelectric layer directly modulates the carrier type and density in the 2D channel, enabling non‐destructive and reversible electrostatic doping. Typically, downward polarization induces electron accumulation (n‐type doping), whereas upward polarization promotes hole injection (p‐type doping), offering a straightforward yet effective mechanism for non‐volatile polarity control. Such polarity programmability allows the same device to be reconfigured into distinct electronic states without altering its physical structure, offering a powerful strategy for multifunctional integration. By spatially programming ferroelectric domains with upward and/or downward polarization states, diverse junction configurations, including p‐n, n‐p, n‐n, and p‐p, can be realized. This concept has been experimentally validated; for instance, lateral homojunctions in MoTe_2_ channels have been arbitrarily formed and reconfigured using scanning probe techniques to locally switch the polarization of P(VDF‐TrFE) layers deposited atop the channel.^[^ ^72^ ^]^ These reconfigurable junctions exhibit pronounced rectification, switchable polarity, and tunable optoelectronic responsivity, highlighting the versatility of ferroelectric gating for programmable logic applications (Figure 2c).

Building upon these demonstrations of domain‐engineered junctions, more advanced device architectures such as dual‐gate or split‐gate FeFETs have been developed to further decouple logic programming from signal modulation, enabling higher‐order logic operations and enhanced functional integration.^[^ ^68^, ^73^ ^]^ In these designs, one gate is dedicated to programming the ferroelectric polarization state—and thus defining the channel polarity—while the other gate governs the ON/OFF switching of the device. This functional separation not only minimizes interference between logic programming and signal processing but also introduces new opportunities for polymorphic logic design. For example, dual‐gate FeFETs allow the realization of reconfigurable logic functions such as AND, OR, and XNOR within a single transistor, significantly reducing circuit complexity and power consumption compared to conventional CMOS approaches. This multifunctionality is exemplified by the reconfigurable logic transistor reported by Ionescu et al., which integrates a Si:HfO_2_ ferroelectric gate with a WSe_2_/SnSe_2_ 2D heterojunction.^[^ ^74^ ^]^ The device incorporates an internal metal electrode in combination with a bottom gate, enabling precise electrostatic control of the semiconducting channel under both ferroelectric and non‐ferroelectric gating modes. Within this single platform, four distinct logic operations are achieved, namely 2D MOSFETs, 2D/2D tunnel FETs, 2D negative‐capacitance (NC) FETs, and NC 2D/2D tunnel FETs. Noteworthy, the NC FET have demonstrated a minimum subthreshold swing (SS) of 29 mV dec^−1^, and the NC tunnel FETs further push this limit to 10 mV dec^−1^, underscoring their potential for ultra‐low‐power operation. Beyond logic switching, the shared ferroelectric gate stack also facilitates neuromorphic functionality, including STDP and pulse‐tunable synaptic weight modulation, co‐integrated on the same WSe_2_ flake. Altogether, this reconfigurable 2D/ferroelectric platform exemplifies how steep‐slope switching, reconfigurable logic, and neuromorphic operations can be seamlessly integrated on a single material platform. Such convergence of logic and neuromorphic functions highlights a pathway toward energy‐efficient, functionally dense computing systems, particularly suited for next‐generation edge intelligence where compactness, adaptability, and low‐power operation are indispensable.

FeFET‐based reconfigurability also extends into the optoelectronic domain. A representative example is demonstrated using ferroelectric‐defined reconfigurable 2D photodiode arrays.^[^ ^75^ ^]^ In this system, a MoTe_2_ homojunction is locally controlled by polarization states of a split‐gated P(VDF‐TrFE) dielectric, producing programmable p‐n or n‐p junctions. These junctions yield oppositely directed built‐in fields and positive/negative photoresponsivities that can be gradually and reversibly tuned via voltage pulses, mimicking LTP and LTD processes. The linear, symmetric modulation of responsivity further enables multiply‐and‐accumulate operations during image acquisition. Experimental demonstrations have shown such photodiodes executing image classification and robotic control tasks without any external memory or computing units, underscoring the potential for unified logic, sensing, and memory within a single hardware layer. These advances collectively underscore the transformative potential of FeFET‐based reconfigurable transistors as a scalable alternative to conventional CMOS scaling. During inference, no external energy consumption occurs at the sensor‐array level due to the device's self‐powered and non‐volatile operation, while the programming energy is as low as 10^−13^ J per operation, comparable to that of bioinspired visual systems (≈10^−15^–10^−13^ J).^[^ ^76^ ^]^ By uniting non‐volatility, field‐programmable logic, and compatibility with emerging 2D semiconductors, FeFETs offer a unique pathway toward ultra‐compact, functionally dense, and adaptive electronics. The convergence of materials innovation, especially in vdW heterostructures and ferroelectric thin films, with architectural breakthroughs such as split‐gate and dual‐gate designs further amplifies their versatility. Moving forward, the development of reconfigurable FeFETs that integrate logic, memory, and sensing into minimal device footprints will be instrumental for next‐generation edge intelligence, where on‐chip adaptability, energy efficiency, and multifunctionality are no longer optional, but essential.

### 2.4 NC‐FETs

In contrast to memory applications that exploit the bistable polarization states of ferroelectrics, NC‐FETs leverage metastable polarization states to overcome fundamental energy‐efficiency limitations in logic scaling. A key performance indicator for NC‐FETs is the SS, defined as the gate voltage required to modulate the drain current by one order of magnitude. In conventional MOSFETs, SS is thermodynamically limited to 60 mV dec^−1^ at room temperature—the so‐called Boltzmann tyranny—which fundamentally impedes further power reduction. By incorporating a ferroelectric layer into the gate stack, NC‐FETs circumvent this limitation. During polarization switching, the ferroelectric material exhibits negative differential capacitance, enabling internal voltage amplification. This voltage amplification enhances the surface potential beyond the applied gate bias, theoretically allowing sub‐60 mV dec^−1^ operation and ultra‐low‐voltage switching.

Recent advancements have demonstrated the feasibility of NC‐FETs across a variety of material systems. Initial studies confirmed that stabilized NC can be realized in ferroelectric/dielectric stacks. For example, MoS_2_ NC‐FETs with HZO/Al_2_O_3_ gate stacks achieved hysteresis‐free sub‐thermionic SS below 1 V through optimized capacitance matching and interface engineering, confirming electrostatic stabilization of the NC state (Figure 2d).^[^ ^77^, ^78^ ^]^ A simplified strategy was later proposed employing a single‐layer polycrystalline PZT gate in MoS_2_ NC‐FETs, where domain‐wall‐driven metastable polarization states enabled sub‐10 mV dec^−1^ SS and ultra‐low‐voltage switching, thereby eliminating the need for additional dielectrics and reducing fabrication complexity.^[^ ^18^ ^]^ Beyond oxide ferroelectrics, vdW ferroelectrics have expanded the material landscape by providing atomically sharp, dangling‐bond‐free interfaces with 2D semiconductors. A notable example is the MoS_2_/CIPS NC‐FET that achieved hysteresis‐free switching with a minimum SS of 28 mV dec^−1^. Importantly, the device also demonstrated retained performance under mechanical bending, underscoring the promise of vdW NC‐FETs for both ultra‐low‐power and flexible applications.^[^ ^79^ ^]^ Parallel efforts with ferroic oxide superlattices demonstrated that HfO_2_‐ZrO_2_ superlattices can sustain mixed ferroelectric‐antiferroelectric ordering down to ≈2 nm, advancing the feasibility of NC‐FETs at sub‐5 nm technology nodes.^[^ ^80^ ^]^ Beyond digital logic, NC has been exploited in multifunctional device platforms. For instance, an HZO‐gated MoS_2_ phototransistor coupled NC with photogating effect is demonstrated capable of achieving few‐photon detection,^[^ ^81^ ^]^ while a WSe_2_ NC‐FET biosensor with Al_2_O_3_/HZO bilayer gates enabled rapid and ultrasensitive glucose detection.^[^ ^82^ ^]^ To illustrate the reproducibility, NC‐FETs employing a 3.0 nm‐thick HZO gate layer subjected to up to 10^10^ switching pulse cycles showed that the SS slightly decreased in the initial cycles but gradually increased with prolonged switching, originating from capacitance mismatching caused by cycling‐induced variations in ferroelectric capacitance.^[^ ^83^ ^]^ Reducing the HZO thickness to 1.5 nm enabled stable polarization up to 10^15^ pulse cycles, underscoring the improved endurance and scalability of ultrathin ferroelectric films.^[^ ^84^ ^]^ Together, these advances underscore the potential of NC‐FETs not only for steep‐slope and energy‐efficient logic, but also as versatile platforms bridging computation, sensing, and flexible electronics.

Despite these encouraging demonstrations, the physical origin and stability of NC remain subjects of ongoing debate. On the one hand, direct experimental evidence supports the existence of intrinsic NC behavior. For example, Yadav et al. provided atomic‐resolution mapping of local NC states within SrTiO_3_/PbTiO_3_ superlattices using electron microscopy, combined with phase‐field and first‐principles simulations. Their results unambiguously visualized NC regions at domain walls with suppressed polarization and elevated energy density.^[^ ^85^ ^]^ Complementarily, Hoffmann et al. showed in Hf_0.5_Zr_0.5_O_2_/Al_2_O_3_ heterostructures that NC behavior is inherent to the ferroelectric layer and independent of domain structure or film thickness.^[^ ^86^ ^]^ On the other hand, numerous studies have shown that apparent NC signatures can also arise from extrinsic or transient effects, complicating the interpretation of steep‐slope behavior. For instance, Wu et al. reported that even in ferroelectric‐free WS_2_ FETs, apparent sub‐60 mV dec^−1^ slopes can be induced purely by gate‐voltage sweep dynamics, indicating that NC‐like signatures may originate from capacitive charging artifacts rather than intrinsic ferroelectric effects.^[^ ^87^ ^]^ In line with this concern, Toriumi et al. observed discrete internal potential jumps near the coercive voltage inferroelectric/paraelectric stacks, attributed to bound‐charge emission during successive domain flips. While these steps can couple to a MOSFET and yield steep SS, they arise from extrinsic switching dynamics instead of a stabilized NC state.^[^ ^88^ ^]^ Expanding on this, Rodder et al. reported that steep SS in HfZrO/SiO_2_‐based FeFETs are governed by delayed domain switching, a transient NC effect tied to polarization dynamics rather than steady‐state NC.^[^ ^89^ ^]^ Additional support comes from HfYO_x_‐based NC‐FETs, where sub‐60 mV·dec^−1^ SS degraded rapidly with cycling due to charge trapping, underscoring its transient nature.^[^ ^90^ ^]^ Complementary studies on epitaxial BaTiO_3_ films also revealed that voltage dips—often interpreted as NC—can be explained by reverse domain nucleation and incomplete charge compensation, with the effect vanishing within a few cycles.^[^ ^91^ ^]^ Taken together, these results indicate that sub‐60 mV dec^−1^ SS alone does not constitute definitive evidence of stabilized NC, and the fundamental question remains whether NC is an intrinsic material property or an emergent phenomenon governed by domain‐wall dynamics and interfacial charge redistribution.^[^ ^92^ ^]^ While theoretical models predict sub‐thermionic SS through internal voltage amplification, device‐level implementation remains challenged by parasitic capacitances and the large quantum capacitance inherent in ultra‐scaled channels, which suppress the effective voltage gain. Consequently, it is anticipated that only device platforms operating near the quantum‐capacitance limit, such as low‐dimensional semiconductors, are expected to fully exploit the potential of NC effect.^[^ ^93^ ^]^

To advance NC‐FETs toward technological adoption, three critical issues must be addressed. First, rigorous and standardized characterization protocols are required to distinguish stabilized NC from transient effects, as experimental artifact such as voltage sweep rate or hysteresis masking can obscure true responses. Second, while most existing models adopt oversimplified single‐domain assumptions and neglect electrostatic inhomogeneity, realistic physical models must incorporate ferroelectric domain dynamics, interfacial effects, and coupled charge transport in the semiconductor channel under realistic boundary conditions. Third, deeper insight into the intrinsic origins of NC, especially through epitaxial films and ab initio calculations, is required to decouple material‐intrinsic phenomena from device‐level limitations. Ultimately, integrating advances in characterization, modeling, and materials engineering will be pivotal to unlocking the full potential of NC‐FETs for next‐generation, low‐power, high‐performance logic technologies.

### 2.5 Multifunctional FeFETs

The intrinsic coupling between ferroelectric polarization and other fundamental degrees of freedom, such as lattice deformation, charge transport, optical excitation, and even quantum states, endows FeFETs with exceptional adaptability to external stimuli. Unlike conventional transistors confined to purely electrical operations, FeFETs respond dynamically to electric fields, mechanical stress, light, and magnetic inputs, giving rise to a variety of cross‐coupled effects, including piezoelectric, electro‐optic, magnetoelectric, piezomagnetic, and magneto‐optic responses. These phenomena enable the seamless integration of multiphysical functionalities into a single device architecture, thereby unlocking new paradigms in multifunctional electronics.

This section explores how such multiphysical coupling mechanisms are strategically harnessed to enable multifunctional behaviors in FeFETs, with particular emphasis on polarization‐engineered photodetection in 2D FeFETs, light‐induced polarization switching in FeSFETs, and multiphysical ferroelectrics for multifunctional FeFETs.

#### 2.5.1 Polarization‐Engineered Photodetection in 2D FeFETs

2D semiconductors exhibit unique photonic and optoelectronic properties arising from their diverse electronic structures and tunable bandgaps. When coupled with ferroelectric materials, their optical performance can be dynamically modulated by the external polarization fields, with FeFETs serving as a representative platform. In this architecture, ferroelectric polarization reconfigures the interfacial charge distribution, which in turn modifies the band alignment of the semiconductor channel. This band‐structure modulation tailors carrier injection barriers and creates strong internal fields that drive the separation of photoexcited electrons and holes. As a result, recombination is suppressed and carrier lifetimes are prolonged, leading to enhanced photocarrier collection efficiency. Collectively, such polarization engineering broadens spectral response, suppresses dark current, and reduces energy consumption. A representative example is the MoS_2_/P(VDF‐TrFE) heterostructure, where ferroelectric polarization generates an ultrahigh local electrostatic field on the MoS_2_ surface—far exceeding that produced by conventional gate voltages.^[^ ^94^ ^]^ This strong modulation enhances carrier mobility and enables broadband photoresponse from the visible regime to 1.55 µm, achieving a high sensitivity of 346.24 AW^−1^ under 20 nW illumination at 450 nm.

Building on these demonstrations, the integration of anisotropic 2D semiconductors introduces additional degrees of freedom for multi‐dimensional optical information processing. A notable achievement is the black phosphorus (BP) homojunction photodetector, where a PN junction is dynamically defined by oppositely polarized P(VDF‐TrFE) domains, yielding an ultra‐sensitive polarization‐dependent photoresponse (Figure 2e). Under ferroelectric modulation, the anisotropic band dispersion of BP is reshaped, leading to a pronounced increase in photothermoelectric current along the armchair direction. Moreover, the ferroelectrically defined PN junction further promotes photothermoelectric current generation and accelerates carrier separation. As a result, the BP photodetector achieves an ultrahigh polarization ratio of 288 at 1450 nm incident light, together with a photoresponsivity of 1.06 AW^−1^ and detectivity of 1.27 × 10^11^ cm Hz^1/2^ W^−1^ at room temperature.^[^ ^95^ ^]^

Beyond discrete photodetectors, polarization‐sensitive FeFETs have also been integrated into in‐sensor artificial neural networks, where signal acquisition and analog preprocessing occur directly within the sensing unit. A notable demonstration involves a ferroelectric photosensor network composed of epitaxial PZT‐based devices, each exhibiting reconfigurable photovoltaic responses with tunable magnitude controlled by ferroelectric polarization.^[^ ^96^ ^]^ This tunability allows each photosensor to act as a signed synaptic weight, enabling direct multiply‐accumulate operations between an input optical image and a photoresponsivity matrix when wired into a network. The system further demonstrates real‐time image processing capabilities, including 100% accurate binary pattern classification and edge detection with an F‐measure of 1, all achieved under self‐powered and zero‐inference‐energy operation, underscore the transformative role of FeFETs in enabling low‐latency, energy‐efficient machine vision systems.

Taken together, these advances illustrate that polarization engineering in FeFETs is not merely a device‐level variable but a scalable design paradigm. At the material and device level, it tailors carrier dynamics to realize broadband photodetection in isotropic semiconductors such as MoS_2_. At the functional level, it exploits anisotropic band dispersions in BP for polarization‐resolved sensing. At the system level, it orchestrates collective behavior in in‐sensor neural networks for real‐time visual processing. Such cross‐scale versatility highlights the potential of FeFETs to unify sensing, memory, and computation within compact, energy‐efficient optoelectronic platforms.

#### 2.5.2 Light‐Induced Polarization Switching in FeSFETs

2D semiconducting ferroelectrics have emerged as promising platforms for optoelectronic applications, owing to their ability to sustain robust ferroelectricity down to atomic thickness and tunable direct bandgaps.^[^ ^97^, ^98^ ^]^ A representative example is In_2_Se_3_, notable for its moderate bandgap of ≈1.46 eV, offering ideal characteristics for integration into nanoscale, ultra‐sensitive photodetectors.^[^ ^99^ ^]^ The spontaneous polarization in α‐In_2_Se_3_ originates from symmetry breaking in its layered crystal structure, where the displacement of the central Se atomic layer induces an upward or downward polarization state. Notably, due to the inherent imprint field arising from interfacial defects, the downward polarization state of α‐In_2_Se_3_ is energetically favored, facilitating non‐volatile polarization retention even in the absence of external fields.

Beyond conventional electric‐field‐induced switching, photoferroelectric effects in α‐In_2_Se_3_ allow for optical modulation of polarization states. Under illumination, the energy barrier separating bistable polarization states is substantially reduced, facilitating spontaneous relaxation from the metastable upward state to the energetically favored downward state. This enables fast, localized, and contactless polarization modulation, which has been harnessed in photoferroelectric FeSFETs that exhibit dual‐mode gating capabilities under both electrical and optical stimuli. Such devices achieve record‐breaking performance metrics—including a noise equivalent power of 7.9 × 10^−22^ W·Hz^−1/2^ and a specific detectivity of 6.3 × 10^17^ Jones.^[^ ^100^ ^]^

More impressively, when implemented in neuromorphic architectures, α‐In_2_Se_3_‐based photoferroelectric FeSFETs demonstrate entangled ferroelectric‐semiconducting and electro‐photonic behavior, enabling simultaneous broadband light sensing and synaptic learning functionality within a single device (Figure 2f).^[^ ^101^ ^]^ The devices have demonstrated retina‐like light adaptation across a wide spectral range of 275–808 nm, with a dynamic range of 20.3 stops, far exceeding the adaptation capability of the human retina. Simultaneously, they support linearly programmable long‐term plasticity, making them ideal for implementing neuromorphic learning tasks. In a fully integrated neuromorphic machine vision system, these FeSFETs have been demonstrated to function both as front‐end retinomorphic sensors and back‐end convolutional neural network nodes. This monolithic system achieved a 93% classification accuracy in a broadband, low‐light image recognition task, being 20% higher than comparable systems lacking the adaptive sensor front‐end. These results underscore the enormous potential of photoferroelectric FeSFETs for realizing highly integrated, low‐power, and adaptive electronic systems that mimic biological perception and cognition.

#### 2.5.3 Multiphysical Ferroelectrics for Multifunctional FeFETs

In the evolving landscape of multifunctional devices, an innovative approach involves the use of channel or dielectric materials that intrinsically respond to multiple external stimuli. Among these, organic photochromic molecules—such as spiropyran, azobenzene, diarylethene, and salicylideneaniline—have long been studied for their light‐induced geometric isomerization and dipole reconfiguration. When incorporated into ferroelectric matrices, these molecular switches offer a promising route for optical control of polarization states through photoisomerization‐driven structural phase transitions. While the photo‐polarization mechanisms discussed in previous sections, such as light‐assisted screening in 2D semiconductors or photoferroelectric switching in ferroelectric semiconductors, rely primarily on photoexcited carrier dynamics and internal field modulation, these photochromic systems introduce a fundamentally different approach by enabling light‐induced intrinsic structural transformations. A significant breakthrough was the synthesis of a series of single‐component organic photochromic ferroelectrics, such as 3,4,5‐trifluoro‐N‐(3,5‐di‐tert‐butylsalicylidene)aniline, which exhibits reversible polarization switching via a light‐triggered enol‐to‐keto structural transformation.^[^ ^102^, ^103^, ^104^, ^105^ ^]^

Beyond established optical responsiveness, the frontier of multi‐stimuli‐responsive ferroelectrics continues to advance. For instance, homochiral organic single‐component crystals such as N‐(3,5‐di‐tert‐butylsalicylidene)‐1‐(4‐bromophenyl)ethylamine represent a new class of photochromic multiferroics exhibiting reversible ferroelectric–ferroelastic phase transitions under light illumination.^[^ ^106^ ^]^ These transitions occur between the enol and trans‐keto forms, enabling dynamic modulation of both spontaneous polarization and strain. Remarkably, these materials support multiple ferroic orders and can be simultaneously tuned by electric fields, mechanical stress, and light, thus representing a true multi‐stimuli‐responsive system. Their low acoustic impedance and fully organic nature position them as excellent candidates for soft electronics, multichannel data storage, and biointegrated optoelectronic systems. Extending this paradigm, molecular multiferroics that simultaneously possess electric dipoles and magnetic anisotropy offer a pathway toward dual‐mode memory devices, such as electric‐write/magnetic‐read architectures. Simulations of Co(NH_3_)_4_N@SWCNT heterostructures have validated this concept, highlighting their promise for next‐generation, low‐energy, and multifunctional memory paradigms.^[^ ^107^, ^108^ ^]^

In addition to these classical physics domains, recent advances have uncovered opportunities for quantum‐ferroelectric coupling in emerging materials.^[^ ^109^ ^]^ Specifically, the coexistence of ferroelectricity and superconductivity in MoTe_2_ offers an unprecedented degree of freedom, enabling reconfigurable superconducting devices capable of serving as magnetic sensors, photon detectors, and superconducting quantum bits (qubits).^[^ ^110^ ^]^ More broadly, such quantum‐ferroelectric interplay opens new directions for FeFET development in cryogenic and multi‐physics regimes, bridging quantum electronics with programmable logic.

Although the integration of such multiphysical materials into fully functional FeFET architectures remains at an early stage, these advances represent pivotal milestones toward the realization of FeFETs with multiphysical responsiveness. Moving forward, the strategic coupling of ferroelectric order with multiple external physical degrees of freedom holds immense promise for building adaptive transistor platforms with unprecedented capabilities in heterogeneous computing, neuromorphic perception, secure memory storage, and quantum‐classical hybrid systems, charting a new path for device innovation at the convergence of materials science and programmable electronics. This section has highlighted the expanding functionality of 2D FeFETs, evolving from non‐volatile memories with efficient data retention, to neuromorphic synapses capable of emulating biological plasticity, to reconfigurable and negative‐capacitance transistors that address the scaling and energy‐efficiency limits of CMOS. Extending beyond electrical operations, FeFETs integrated with 2D semiconductors, photoferroelectric materials, molecular switches, and quantum systems demonstrate multifunctional responses to optical, mechanical, and quantum stimuli. Together, these advances establish ferroelectric polarization as a unifying principle enabling tunable interactions across material, device, and system levels. By strategically harnessing this coupling, FeFETs provide a coherent pathway toward adaptive, compact, and energy‐efficient platforms, with potential applications ranging from heterogeneous computing and in‐sensor intelligence.

## 3 Integration of Ferroelectric Domains at (Sub)Nanometer Scale

Realizing ultrahigh‐density data storage requires pushing ferroelectricity to the edge of stability by identifying the minimum structural dimensions that can sustain switchable polarization. Distinct ferroelectric systems—including perovskite oxides, hafnia‐based oxides, fluoropolymers, vdW ferroelectrics, and molecular ferroelectrics—exhibit diverse origins of polarization. These underlying mechanisms determine their key parameters, such as remanent polarization, coercive field, and scalability, thereby governing how ferroelectricity can be retained at the (sub)nanometer limit. Understanding the fundamental relationship between material class and polarization mechanism is thus essential for guiding device miniaturization and integration.

This section discusses the fundamental mechanisms that enable or hinder polarization retention at the (sub)nanometer scale in each material class, highlighting both their intrinsic limits and opportunities for device miniaturization. **Table** **2** summarizes these critical dimension thresholds across representative ferroelectric systems, providing a comparative overview of their scale‐dependent behaviors.

**Table 2:** Summary of the critical size required to maintain ferroelectricity in representative ferroelectric materials. Reproduced with permission.^[^ ^80^ ^]^ Copyright 2022, Springer Nature. Reproduced with permission.^[^ ^97^ ^]^ Copyright 2016, Springer Nature. Reproduced with permission.^[^ ^125^ ^]^ Copyright 2019, Springer Nature. Reproduced with permission.^[^ ^129^ ^]^ Copyright 2017, Springer Nature. Reproduced with permission.^[^ ^126^ ^]^ Copyright 2018, Springer Nature. Reproduced with permission.^[^ ^132^ ^]^ Copyright 2024, The American Association for the Advancement of Science. Reproduced with permission.^[^ ^135^ ^]^ Copyright 2021, Springer Nature. Reproduced with permission.^[^ ^141^ ^]^ Copyright 2023, Springer Nature. Reproduced with permission.^[^ ^144^ ^]^ Copyright 2023, Springer Nature. Reproduced with permission.^[^ ^147^ ^]^ Copyright 2023, Springer Nature. Reproduced with permission.^[^ ^148^ ^]^ Copyright 2016, American Chemical Society. Reproduced with permission.^[^ ^149^ ^]^ Copyright 2017, Springer Nature. Reproduced with permission.^[^ ^150^ ^]^ Copyright 2021, Springer Nature. Reproduced with permission.^[^ ^151^ ^]^ Copyright 2018, The American Association for the Advancement of Science. Reproduced with permission.^[^ ^153^ ^]^ Copyright 2023, American Chemical Society. Reproduced with permission.^[^ ^108^ ^]^ Copyright 2023, American Chemical Society.

Classification		Representative materials	Structure	Critical size maintaining ferroelectricity	Origin	Refs.
Perovskite oxide		BiFeO_3_	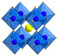	2.4 nm single‐unit‐cell	Ionic displacement	[113, 114]
Hafnia‐based oxides		HfO_2_/ZrO_2_	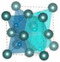	0.5 nm single‐unit‐cell	Combination of thermodynamic and kinetic factors	[116, 117, 118, 119, 121]
Fluoropolymer		_P(VDF‐TrFE)_	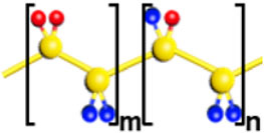	1 nm	Polar molecular groups	[124]
VdW	Intrinsic	CIPS	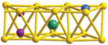	monolayer	Ionic displacement	[97]
		d1T‐MX_2_ (M = Mo, W; X = S, Se, Te)	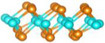	monolayer	Ionic displacement	[125]
		MX (M = Ge, Sn; X = S, Se, Te)	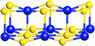	monolayer	Ionic displacement	[127, 128]
		In_2_Se_3_	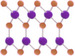	monolayer	Ionic displacement	[129]
		WTe_2_	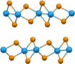	two atomic layers	Charge‐redistribution	[126]
		BP‐Bi	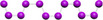	monolayer	Ionic displacement & charge redistribution	[130]
	Interlayer sliding	homobilayers h‐BN	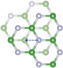	two atomic layers	Charge‐redistribution	[131, 132]
		r‐stacked MX_2_ (M = Mo/W, X = S/Se)	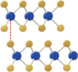	two atomic layers	Charge‐redistribution	[133]
		heterobilayers MoS_2_/WS_2_	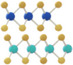	two atomic layers	Charge‐redistribution	[134]
	Moiré	homobilayers h‐BN, graphene	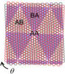	two atomic layers	Charge‐redistribution	[135, 136, 137, 138, 139, 140]
		heterobilayers WTe_2_/WSe_2_	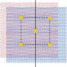	two atomic layers	Charge‐redistribution	[141, 142, 143]
	Intralayer sliding	GaSe	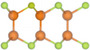	monolayer	Ionic displacement	[144]
1D		Te nanowires	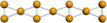	30 nm × 5 nm	Ionic displacement	[146]
		WS_2_ nanotubes	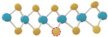	< 10 nm × 10 nm × 2 nm	Ionic displacement	[147]
Molecular ferroelectrics		Ionic crystals [(CH_3_)_2_CH_2_)_4_N]ClO_4_	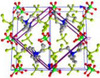	/	Ionic‐displacement	[148]
		Supramolecular complexes Cu(1,10‐phenlothroline)_2_SeO_4_· (diol)	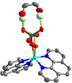	/	Ionic‐displacement	[149]
		Hybrid organic‐inorganic perovskites [(CH_3_)_2_CHCH_2_NH_3_]_2_PbCl_4_	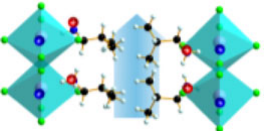	/	Ionic‐displacement	[150]
		Metal‐free perovskites ABX_3_ (A = divalent organic ammonium cation, B = NH_4_^+^, X = I/Br/Cl)	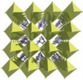	/	Ionic‐displacement & polar molecular groups	[151]
		Fullerenes C_60_S_8_	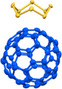	/	Polar molecular groups	[153]
		Multiferroics Co(NH_3_)_4_N@SWCNT	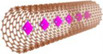	/	Polar molecular groups	[108]

Materials with the perovskite structure ABO_3_ have long served as the prototypical ferroelectrics, where A and B represent two distinct metal elements. Their spontaneous polarization arises from the relative displacement of the central ion within the oxygen octahedron, typically moving toward one of the surrounding O^2−^ ions. The resulting dipole moment defines the polarization direction from the negative to the positive charge center.^[^ ^111^ ^]^ However, perovskite ferroelectrics often suffer polarization loss due to depolarization effects at reduced dimensions.^[^ ^111^, ^112^ ^]^ Nonetheless, advances in thin‐film fabrication have enabled freestanding perovskite films, such as BiFeO_3_, to retain ferroelectricity down to nearly single‐unit‐cell limit.^[^ ^113^, ^114^ ^]^

The discovery of ferroelectricity in Si‐doped HfO_2_ thin films has spurred extensive research for this material.^[^ ^115^ ^]^ Since amorphous HfO_2_ has already been maturely utilized as a high‐k gate dielectric in CMOS devices, ferroelectric HfO_2_ can be seamlessly integrated into mainstream semiconductor manufacturing platforms. Remarkably, they have been demonstrated to maintain switchable polarization down to the angstrom scale, even when integrated with silicon platforms.^[^ ^116^, ^117^ ^]^ However, the intricate nature of this unconventional robust ferroelectricity remains a topic of debate, primarily due to the complex coexistence of multiple phases within the material. First‐principal calculations suggest that flat phonon bands in HfO_2_ give rise to stable, independently switchable dipoles that are resilient to extrinsic perturbations.^[^ ^118^ ^]^ Complementary studies further highlight the critical role of reversible oxygen migration and phase transitions in stabilizing ferroelectric properties,^[^ ^119^ ^]^ as well as the significant influence of surface electrochemical states.^[^ ^120^ ^]^ These insights are applicable to other binary ferroelectrics such as ZrO_2_, which sustains ferroelectricity at 5 Å—equivalent to its unit‐cell size, offering opportunities for single‐unit‐cell FeFETs.^[^ ^121^ ^]^

Compared with the rigid ferroelectric oxides, organic ferroelectric polymers generally exhibit lower spontaneous polarization, higher coercive fields, and limited environmental stability.^[^ ^122^ ^]^ Nonetheless, they offer distinct advantages such as mechanical flexibility, biocompatibility, and low crystallization temperatures, making them highly attractive for flexible and wearable electronics.^[^ ^123^ ^]^ Among ferroelectric polymers, poly(vinylidene fluoride) (PVDF) and its derivatives represent the most extensively studied systems. The C─F and C─H bonds in PVDF generate strong electric dipoles, where the high electronegativity of F atom drives the electron cloud toward its side, creating localized negative charge and enabling switchable polarization along the polymer chain. Particularly, monolayer P(VDF‐TrFE) films fabricated via Langmuir–Blodgett deposition exhibit intrinsic ferroelectricity, further underscoring the feasibility of molecular‐scale polarization retention.^[^ ^124^ ^]^

Progress in vdW ferroelectric materials has extended the retention of ferroelectricity down to the 2D limit. Depending on the polarization orientation, ferroelectricity in vdW materials can be categorized as either out‐of‐plane or in‐plane, referring to polarization directions perpendicular or parallel to the atomic plane, respectively. Intrinsic out‐of‐plane ferroelectricity has been experimentally verified in CuInP_2_S_6_ (CIPS),^[^ ^97^ ^]^ d1T MoTe_2_^[^ ^125^ ^]^ and WTe_2_,^[^ ^126^ ^]^ and in‐plane ferroelectricity in group‐IV monochalcogenides MX (M = Ge, Sn; X = S, Se),^[^ ^127^, ^128^ ^]^ In_2_Se_3_^[^ ^129^ ^]^ and single‐element Bi.^[^ ^130^ ^]^ While owing to the strict lattice symmetry constraints for the ferroelectricity preservation, such intrinsic vdW ferroelectrics are relatively rare. Sliding ferroelectricity—driven by interlayer displacements—offers alternative routes to engineer polarization in otherwise centrosymmetric bilayers. It has been demonstrated in homobilayers like h‐BN,^[^ ^131^, ^132^ ^]^ rhombohedral (r)‐stacked MX_2_(M = Mo/W, X = S/Se),^[^ ^133^ ^]^ as well as heterobilayers like MoS_2_/WS_2_,^[^ ^134^ ^]^ Moiré‐engineered bilayers, such as h‐BN,^[^ ^135^, ^136^ ^]^ graphene,^[^ ^137^, ^138^ ^]^ WSe_2_,^[^ ^139^ ^]^ MoS_2_,^[^ ^140^ ^]^ WTe_2_/WSe_2_,^[^ ^141^ ^]^ MoSe_2_/WSe_2_,^[^ ^142^ ^]^ and WSe_2_/BP,^[^ ^143^ ^]^ further enrich this landscape. Furthermore, intralayer sliding ferroelectricity has been demonstrated in materials like GaSe.^[^ ^144^ ^]^ Theoretical predictions suggest that interlayer displacement‐induced ferroelectricity could be realized across all 80 crystallographic 2D layer groups.^[^ ^145^ ^]^ These vdW ferroelectrics also exhibit functional attributes such as dipole locking, ferroionic coupling, negative piezoelectricity, and photo enhancement, making them promising for post‐Moore nanoelectronics and optoelectronics.

Beyond the 2D regime, ferroelectricity has been further downscaled into quasi‐1D systems in single‐element materials like Te nanowires, where lone‐pair electron‐mediated ion displacements stabilize polarization along the chain direction.^[^ ^146^ ^]^ Devices based on Te nanowire channels exhibit high carrier mobility (≈220 cm^2^ V^−1^ s^−1^), fast switching (<20 ns), long retention (>10^5^ s), and ultrahigh storage density (>1.9 TB cm^−2^), demonstrating their potential for ultrahigh‐density memory and computing‐in‐memory applications.

Pushing the dimensional scaling to the extreme, 0D ferroelectricity has been demonstrated at vdW interfaces in crossed WS_2_ nanotubes.^[^ ^147^ ^]^ Atomic sliding at the confined interface gives rise to interfacial polarization, enabling the construction of 0D ferroelectric diodes. Such devices transcend conventional scaling limits by uniting non‐volatile resistive switching with programmable photovoltaic responses in the visible spectrum, all within a single nanoscale junction. Their operation at ultralow current densities, dictated by intrinsic dimensional confinement, highlights a unique pathway toward energy‐frugal memory‐photonic integration. Another promising direction lies in molecular ferroelectrics.^[^ ^148^, ^149^, ^150^, ^151^ ^]^ Their nearly unlimited chemical tunability, solution processability, mechanical flexibility, and biocompatibility make them ideal for wearable and ultracompact devices. While bulk ferroelectrics require domain nucleation and growth for polarization switching, isolated molecular ferroelectrics achieve polarization reversal through the flipping of individual dipoles. This independence from neighboring interactions enables faster writing speeds and potentially solves issues like domain instability and fatigue that have hindered the efficiency of traditional FeFET applications. Moreover, the ability for each molecule to encode a single data bit provides a pathway toward molecular‐scale data storage. Pioneering research has predictied the feasibility of endohedral fullerite ferroelectrics as candidates for ultrahigh‐density non‐volatile memories with storage capacities up to approximately 10^5^ Gbit/in^2^, surpassing the 10^2^ Gbit/in^2^ storage capacity of conventional ferroelectrics.^[^ ^152^, ^153^ ^]^ Experimental validation has further bolstered this concept, with gate‐controlled switching behavior demonstrated between two electronic states in a single‐molecule electret device based on Gd@C_82_.^[^ ^154^ ^]^

As the scaling of ferroelectric materials approaches the single‐unit‐cell limit, significant challenges arise in advancing single‐molecule FeFETs. First, the intrinsic uniformity of ferroelectric layers imposes a fundamental limitation in ferroelectric domain engineering. As each unit‐cell ferroelectric layer shares identical local coercive field *E*_c_ and remanent polarization P_r_, the homogeneity leads to collective switching of all the domains once the critical *E*_c_ is reached, precluding selective manipulation at the unit‐cell level. To address this constraint, strategies to deliberately break this uniformity have been proposed. In particular, digitalized lateral gradient doping—realizable through techniques such as pulsed laser deposition, molecular beam epitaxy, or atomic layer deposition—offers a practical route to discretize local *E*_c_ and *P*_r_ across individual layers.^[^ ^155^ ^]^ By engineering spatially varied switching thresholds, discrete unit‐cell‐by‐unit‐cell polarization reversal can be achieved. This enables multi‐level memory operation within a single ferroelectric film, with distinct *E*_c_ values encoding multiple stable storage states. Additionally, conventional ferroelectric switching often requires the application of large biases, which substantially increases the risk of dielectric breakdown and long‐term reliability failure. This issue is particularly acute in vdW ferroelectrics such as CIPS, where field‐driven polarization reversal is intimately coupled with ionic migration, frequently resulting in erratic or even destructive switching. To mitigate this limitation, electric‐field‐free switching strategies are being actively explored. Among these, the flexoelectric switching—where polarization is modulated through mechanical strain gradients—has emerged as a particularly promising route. By leveraging strain‐induced polarization rather than external bias, this approach enables low‐voltage, nondestructive domain control, offering a safer and more energy‐efficient pathway for nanoscale ferroelectric switching.^[^ ^156^, ^157^ ^]^ Finally, achieving angstrom‐scale electrode patterning remains critical for ultrafine domain control. In the ideal case, to fully exploit unit‐cell‐scale switching in FeFETs, line‐type electrodes with sub‐nanometer pitch would be required. In practical, where such angstrom‐scale electrode fabrication remains technologically prohibitive, sparse electrode arrays with nanometer‐scale spacing in line‐cell configurations can still be employed.^[^ ^118^ ^]^ This approach already offers notable improvements in memory density compared with conventional architectures, with further gains dependent primarily on advances in electrode deposition and nanoscale etching technologies. By and large, the integration of ferroelectric domains at the subnanometer scale requires a confluence of material innovation, structural engineering, and advanced processing techniques. The synergistic efforts chart a path toward ultra‐dense, multifunctional devices tailored for the post‐Moore era.

The progressive downscaling of ferroelectric domains from bulk crystals to the (sub)nanometer regime has revealed both the untapped opportunities and intrinsic limits across diverse material classes. The convergence of emerging ferroelectrics, from robust hafnia‐based oxides to molecular systems, charts a pathway toward single‐unit‐cell FeFETs. Realizing ultrahigh‐density ferroelectric devices will require the integration of these material advances with precision domain engineering, interface design strategies, and angstrom‐scale electrode patterning. Such progress not only pushes ferroelectricity to its ultimate scaling limits but also lays the foundation for More Moore‐oriented process innovations, where device miniaturization, fabrication precision, and integration compatibility become coequal priorities alongside functional diversification.

## 4 Embedding Functional Molecular Switches for Function Diversification

While the multiphysical ferroelectrics discussed in the previous section have laid the foundation for multifuctional FeFETs, further diversification requires more modular and programmable approaches. Functional molecular engineering provides such a pathway, with molecular switches standing out for their ability to undergo reversible structural and electronic transformations under external stimuli such as light, heat, or electric fields.

**Figure 3:**
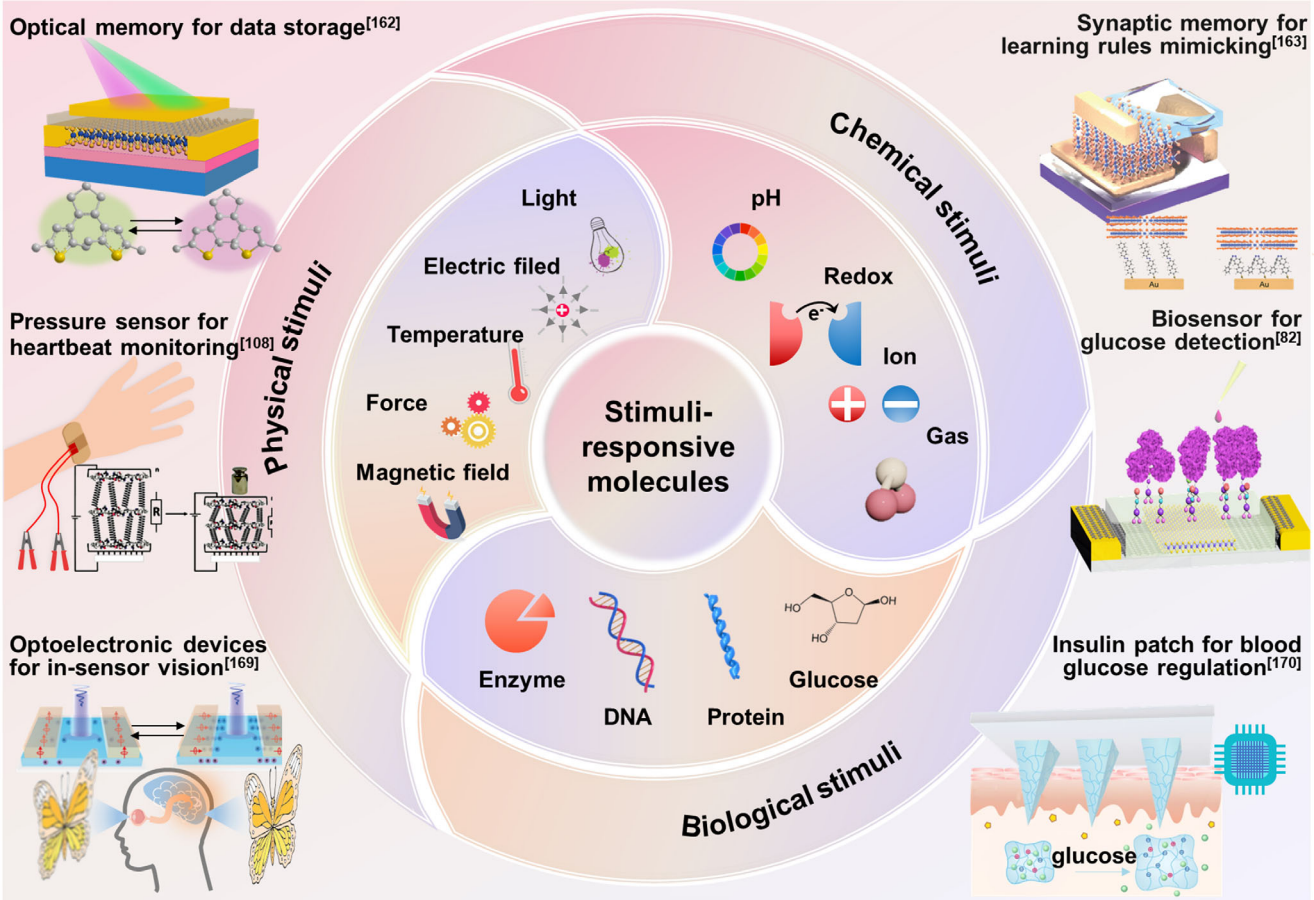
Overview of stimuli‐responsive molecules and representative examples of their application in digital electronics. Left panel: Optical memory for data storage by incorporating photochromic molecules. Reproduced with permission.^[^ ^162^ ^]^ Copyright 2021, Wiley. Pressure sensor for heartbeat monitoring by incorporating strain‐sensitive molecules. Reproduced with permission.^[^ ^168^ ^]^ Copyright 2019, Wiley. Optoelectronic devices for in‐sensor vision by incorporating ferromagnetic molecules. Reproduced with permission.^[^ ^169^ ^]^ Copyright 2024, Wiley. Right panel: Synaptic memory for learning rules mimicking by incorporating redox‐responsive molecules. Reproduced with permission.^[^ ^163^ ^]^ Copyright 2024, Wiley. Biosensor for glucose detection by incorporating enzyme‐responsive molecules. Reproduced with permission.^[^ ^82^ ^]^ Copyright 2024, American Chemical Society. Insulin patch for blood glucose regulation by incorporating a glucose‐responsive polymeric matrix. Reproduced with permission.^[^ ^170^ ^]^ Copyright 2020, Springer Nature.

This section provides an overview of molecular switches integrated into 2D FETs, introducing their fundamental operating principles and associated functional characteristics. The discussion emphasizes how molecular design governs device performance and highlights the key challenges toward practical integration. **Figure** **3** summarizes representative examples of molecular switches and their application in digital electronics.

**Figure 4:**
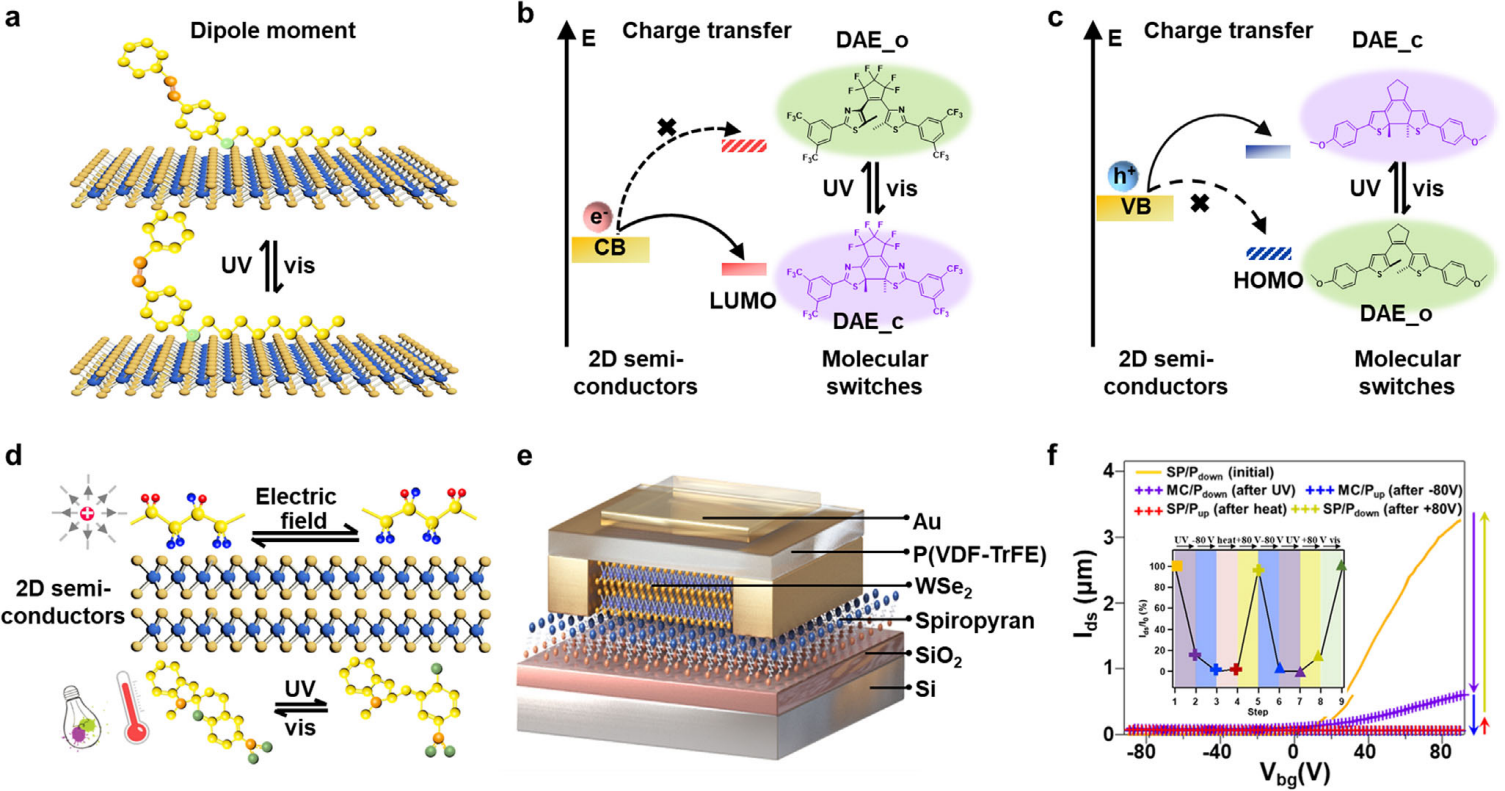
Embedding functional molecular switches into 2D FeFETs. a) Schematic illustration of reservable dipole‐induced doping in 2D semiconductors modulated by azobenzene molecular switches under UV/visible irradiation. Reproduced with permission.^[^ ^158^ ^]^ Copyright 2019, ACS nano. b,c) Energy‐band diagrams showing reversible charge transfer between diarylethene molecules and 2D semiconductors during photoisomerization. Reproduced with permission.^[^ ^160^ ^]^ Copyright 2020, Wiley. d) Schematic of a Janus‐configured 2D semiconductor, where the top surface is interfaced with fluoropolymers and the bottom surface is functionalized with photochromic molecular layers, enabling dual‐field modulation. e) Device schematic of the molecular‐switch‐integrated 2D FeFET. f) Transfer characteristics of the device in (e), showing reversible current modulation under electric field, light, and thermal stimuli. Reproduced with permission.^[^ ^161^ ^]^ Copyright 2021, ACS nano.

The transformations of functional molecules strongly modulate their dipole moments and charge‐transfer characteristics, which in turn reshape the interfacial electrostatic environment. When integrated with 2D semiconductors, whose atomically thin structure is highly sensitive to interfacial perturbations, such molecular‐level dynamics translate into tunable doping levels, band alignments, or local electrostatic environments. Specifically, the orientation of molecular dipoles can generate out‐of‐plane electric fields that shift the Fermi level and modify the work function of the 2D semiconductor, effectively functioning as a built‐in gate bias (**Figure** **4**a). For instance, azobenzene molecules undergo efficient photoisomerization between trans and cis configurations under UV and visible light irradiation, exhibiting diverse dipole moments. When integrated with 2D semiconductors, such dipolar transitions enable reversible modulation of carrier concentration and even carrier polarity of 2D semiconscious through interfacial dipole coupling under light.^[^ ^158^ ^]^ Meanwhile, charge transfer between the molecular layer and the 2D semiconductor can occur when there is favorable alignment between the molecular frontier orbitals and the semiconductor energy levels. If the molecular lowest unoccupied molecular orbital (LUMO) lies below the conduction band (CB) of the 2D semiconductor, electrons can transfer from the semiconductor to the molecule, inducing p‐type doping. Conversely, if the molecular highest occupied molecular orbital (HOMO) lies above the valence band (VB), electron donation from the molecule to the semiconductor leads to n‐type doping (Figure 4b,c). For example, diarylethene‐functionalized WSe_2_ demonstrates photoisomerization‐induced modulation of energy levels: one isomeric state acts as a charge trap, while the other serves primarily as a scattering center, enabling reversible tuning of carrier density under optical excitation.^[^ ^159^, ^160^ ^]^ Through these coupled mechanisms, related 2D FETs can acquire programmable electrical responses dictated by molecular state, including stimulus‐triggered logic gating, neuromorphic adaptability, and environmental sensing.

In the special case of 2D FeFETs, the intrinsic spatial decoupling, where ferroelectric gating occurs on one surface, leaves the opposite surface accessible for molecular functionalization. This dual‐interface configuration enables independent optimization of gate control and stimulus responsiveness, thereby extending the multifunctionality of FeFETs and directly bridges molecular design with device‐level diversification (Figure 4d). Pioneering this approach, Janus‐configured hybrid systems have been assembled by integrating ferroelectric P(VDF‐TrFE) on one facet of few‐layer WSe_2_, while functionalizing the reverse surface with photochromic molecules such as diarylethene or spiropyran (Figure 4e).^[^ ^161^, ^162^, ^163^ ^]^ This dual‐sided architecture enables independent yet synergistic control, where ferroelectric polarization induces electrostatic doping and molecular conformational switching modulates trap states or dipole moments. Through this decoupled electric–optical modulation, the channel conductance can be reversibly tuned by up to 87%—more than twice that of conventional single‐stimulus‐responsive devices (Figure 4f).^[^ ^162^ ^]^ In memory‐related applications, such enhanced modulation translates into increased storage capability through multi‐state programmability. Notably, this modular approach circumvents the need for complex multifunctional molecule synthesis and is readily extendable to diverse combinations of 2D semiconductors and functional molecular layers.

Redox‐switchable molecules represent another important class of active components that endow FETs with dynamic and reversible functionalities. These electrochemically driven molecular switches can undergo reversible oxidation–reduction reactions, yielding distinct and stable electronic configurations that differ in charge distribution. Typical examples include ferrocene, tetrathiafulvalene, viologens, and quinone derivatives, each featuring two or three well‐defined redox states that can be interconverted with high reversibility.^[^ ^164^ ^]^ Redox‐functionalized 2D FETs have been realized by adsorbing ferrocene derivatives onto MoS_2_ channels, where the ferrocene/ferrocenium (Fc/Fc⁺) couple serves as a molecular charge reservoir capable of modulating the carrier density and threshold voltage of the underlying semiconductor.^[^ ^165^ ^]^ This doping effect originates from the electrostatic gating induced by the charged or neutral molecular states. In particular, the oxidized Fc^+^ species accumulate electrons and induce n‐type doping on MoS_2_. Such device architecture offers a versatile strategy to realizing electrochemically switchable 2D FETs, where the redox potential serves as a remote and reversible control signal for tuning electronic transport.

Spin‐switchable molecules represent an important class of active components that enable dynamic control of magnetic properties in 2D FETs. Typical spin‐switchable species include spin‐crossover SCO complexes, organometallic compounds, and single‐molecule magnets.^[^ ^166^ ^]^ A representative example is the quinoidal dithienyl perylenequinodimethane (QDTP), which undergoes a spin transition from a singlet state at low temperature to a triplet state above 370 K.^[^ ^167^ ^]^ When interfaced with 2D materials, this molecular spin transition can be electrically transduced. In graphene–QDTP heterostructures, the switch induces reversible hole doping and a pronounced positive magnetoresistance (≈50%) is observed, whereas in MoS_2_–QDTP devices, electron doping dominates, accompanied by a transition to negative magnetoresistance (≈100%). These findings demonstrate the feasibility of integrating switchable magnetic molecules with 2D semiconductors, enabling electrically addressable spintronic devices capable of high‐temperature spin detection and magnetic state modulation.

Beyond molecular switches, recent advances have expanded molecular control to encompass multi‐physics coupling. Strain‐sensitive molecular systems, such as amino‐functionalized reduced graphene oxide with covalently tethered spacers, have enabled tunable piezoresistive responses and enhanced sensitivity in low‐pressure detection applications.^[^ ^168^ ^]^ Magneto‐photoresponsive materials, exemplified by Fe_3_GaTe_2_ integrated as electrodes with WSe_2_ channels, have facilitated gate‐free architectures for in‐sensor vision and visual adaptation.^[^ ^169^ ^]^ Biological‐responsive systems, including glucose‐sensitive polymer matrices embedded in microneedle patches, have achieved autonomous insulin release under hyperglycemic conditions, offering a promising platform for closed‐loop drug delivery.^[^ ^170^ ^]^ Beyond synthetic molecular switches, sustainable biopolymer‐based interfaces have also been introduced. For instance, sodium alginate incorporated into Janus architectures can act as an opto‐ionic interface on the opposite surface of ferroelectric‐gated 2D semiconductors, leveraging structural decoupling to maximize multifunctionality while promoting eco‐friendly material choices.^[^ ^171^ ^]^ Taken together, these advances illustrate how functional molecular engineering broadens FeFET functionality from single‐stimulus response to multi‐modal programmability, while simultaneously aligning with sustainability imperatives. As Moore's law faces its scaling limits, they shift focus from mere size reduction to functional diversification and cross‐disciplinary integration, signalling a More than Moore trajectory for future semiconductor technologies.

The performance of these molecularly functionalized FeFETs is inherently influenced by the intrinsic properties of the embedded molecules. The interfacial local electrostatic environment, shaped jointly by the dipole moments of the molecular switches or the alignment of their frontier orbitals with the semiconductor band edges, plays a decisive role in governing carrier injection. Favorable dipolar reorientation and band alignment lower injection barriers and enhance charge transfer across the interface, thereby increasing current modulation efficiency. This directly impacts the dynamic range of conductance states in FeFETs and ultimately determines the achievable storage density in memory applications. Besides, long‐term memory reliability requires improvements in fatigue resistance and retention. Fatigue resistance is often compromised by the accumulation of isomerization byproducts during repeated switching cycles, which disrupt reversible pathways. Retention, in turn, depends on the stability of molecular configurations, as metastable isomers tend to relax over time, leading to charge loss and degraded non‐volatility. To address these reliability limitations, molecular optimization strategies have been developed. Diarylethene is a representative case, undergoing photocyclization and cycloreversion between open‐ and closed‐ring isomers under UV and visible light. This configurational switching is accompanied by a shift of their corresponding frontier orbitals, therefore dynamically regulates carrier injection into the interfaced 2D semiconductor. For enhanced modulation efficiency, molecules with larger energy‐level offsets relative to the semiconductor are preferred,^[^ ^172^ ^]^ which can be achieved by chemical functionalization of the aryl rings or ethylene bridge, enabling energy‐level shifts exceeding 2 eV.^[^ ^173^ ^]^ Fatigue resistance, on the other hand, can be improved by suppressing side reactions through substituents like 3,5‐bis(trifluoromethyl)phenyl and 3,5‐bis(pentafluorosulfanyl)phenyl, which reduce byproduct formation and enable reversible cycling.^[^ ^174^ ^]^ Retention can be strengthened by reinforcing thermal bistability, for instance by reducing the aromaticity of the ethene bridge with benzobis(thiadiazole), which stabilizes the closed‐ring isomer and improves robustness.^[^ ^175^, ^176^ ^]^ Moreover, to overcome the intrinsic limitations of UV activation, including photodamage, shallow penetration, and nonselective absorption,^[^ ^177^ ^]^ red‐shifted activation strategies have been developed, such as introducing an aromatic dye at the reactive carbon atom,^[^ ^178^ ^]^ employing sensitizer building blocks with narrow singlet‐triplet energy gaps to a chromophore core,^[^ ^179^ ^]^ and incorporating donor‐acceptor groups at both ends of the chromophore unit to facilitate low‐energy intramolecular charge transfer transition.^[^ ^180^ ^]^ Overall, achieving precise control over the molecular structure and understanding substituent effects are vital for tailoring the functionalities of molecular switches to meet specific device requirements.

Although molecular functionalization offers a powerful route for diversifying FeFET functionalities, its industrial viability remains contentious due to challenges such as insufficient thermal robustness, limited processability, and poor compatibility with standard CMOS integration protocols. The key factor underlying these challenges is the inherent dimensional mismatch between quasi‐0D molecular switches and the laterally extended, atomically thin surfaces of 2D semiconductors. This geometric asymmetry complicates uniform coverage and consistent interaction at the interface, being critical factors for reliable device performance. To address this challenge, two functionalization strategies have been explored. The first involves forming a (semi‐)continuous molecular thin film over the 2D surface, particularly effective for weakly interacting molecules, as dense packing suppresses thermal diffusion and enhances cooperative dipole effects. The morphology and molecular order of the film critically determine the uniformity of doping, dielectric screening, and switching reproducibility. The second strategy focuses on site‐selective anchoring of isolated molecules at specific lattice defects, edge sites, or vacancies, enabling localized modulation of charge density, trap states, or band alignment. This approach is advantageous for strongly binding molecules that resist self‐assembly but can be immobilized at predetermined sites with atomic precision, albeit requiring robust molecule–substrate interactions to remain stable under thermal cycling and electrical stress. Both approaches present a trade‐off between chemical robustness and electronic integrity. While noncovalent adsorption is integration‐friendly and minimally disruptive to the 2D lattice, they suffer from diffusion and unstable doping under high electric fields. In contrast, covalent anchoring ensures better thermal and chemical endurance, they may disrupt the 2D lattice or introduce localized states, thereby deteriorating carrier mobility and increasing scattering at the 2D surfaces. These limitations underscore the need for mild covalent anchoring approaches, such as edge‐selective functionalization^[^ ^181^, ^182^ ^]^ or defect‐site anchoring with tailored linkers,^[^ ^183^, ^184^ ^]^ that minimize disruption to the basal 2D lattice while maintaining chemical robustness under fabrication and operational conditions, offering a practical path toward reliable, molecularly functionalized FeFETs.

Another pressing challenge lies in achieving precise control over device performance and carrier polarity through molecular doping, which is crucial for the development of energy‐efficient and functionally reconfigurable FeFET platforms. A promising strategy involves the rational design of dopants bearing tunable electron‐donating or electron‐withdrawing substituents.^[^ ^185^ ^]^ These functional groups not only induce distinct dipole moments but also govern the spatial orientation of dopants at the ferroelectric/semiconductor interface, thereby modulating local electric fields and tuning band alignment. Beyond chemical design, advanced interface engineering offers additional precision. Spatially controlled selective doping,^[^ ^186^, ^187^ ^]^ enabled by methods such as electron‐beam‐defined area functionalization, nanoimprint lithography, allows localized dopant placement in targeted regions. Such precise control over molecular doping not only facilitates complementary NMOS/PMOS integration, but also enables dynamically reconfigurable FeFET platforms capable of logic switching, analog computing, or memory‐in‐logic operations. This multifunctional versatility is pivotal for addressing the growing demand for adaptive and energy‐efficient electronics in the post‐Moore era.

Such molecular engineering strategies not only endow FeFETs with dynamic, stimulus‐responsive functionalities beyond static switching, but also pave the way for heterogeneous integration of soft‐matter logic layers atop conventional semiconductor platforms, bridging molecular‐level reconfigurability with scalable, CMOS‐compatible architectures for next‐generation adaptive electronics. By enabling precise control over interfacial dipoles, doping profiles, and environmental sensitivity, these hybrid systems allow seamless fusion of electrical, optical, magnetic, and biochemical responsiveness within a single reconfigurable framework. Importantly, the structural decoupling intrinsic to 2D FeFETs offers a unique spatial freedom to independently optimize gate control and functional interface engineering, supporting vertically stackable device architectures and BEOL process integration. Together, these advances chart a promising course toward post‐Moore semiconductor paradigms where functional diversity, energy efficiency, and architectural modularity are prioritized over mere geometric scaling.

## 5 M3D Integration for Enhanced Chip‐Level Density and Functionality

M3D integration has emerged as a promising strategy to address both scaling bottlenecks and the demand for multifunctionality in the post‐Moore era. Unlike traditional 2D layouts, it enables vertical stacking of functional device layers interconnected via dense vertical vias, thereby pushing chip‐level integration density beyond the limits of planar scaling in the spirit of More Moore. At the same time, the heterogeneous integration of distinct device functionalities across stacked tiers introduces new design flexibility and multifunctional capabilities, aligning with the concept of More than Moore. By minimizing the physical distance between sensors and processors, the architecture effectively reduces latency and power consumption, achieving up to a thousand‐fold improvement in the energy‐delay product compared to their 2D counterparts.^[^ ^188^ ^]^ Taken together, M3D integration offers a unifying pathway to pursue both ultrahigh‐density scaling and functional diversification, positioning it as a cornerstone for next‐generation information technologies.

**Figure 5:**
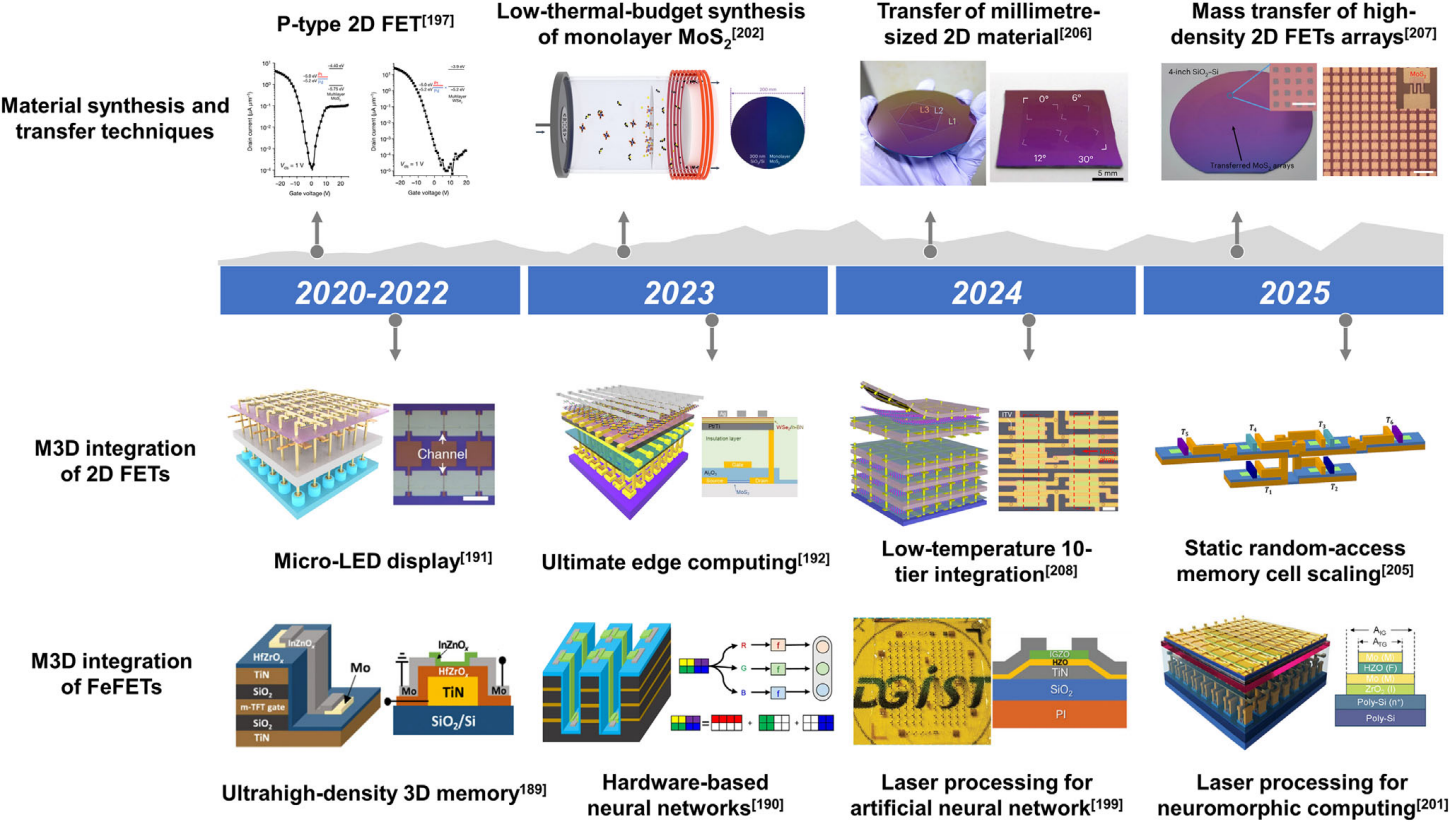
Recent five‐year landmarks in material synthesis and transfer techniques (up panels) and devices demonstrations (below panels) for M3D integration. Up panels: P‐type electrical contacts Pt and Pd for 2D FET. Reproduced with permission.^[^ ^197^ ^]^ Copyright 2022, The American Association for the Advancement of Science. Low‐thermal‐budget synthesis of monolayer MoS_2_. Reproduced with permission.^[^ ^202^ ^]^ Copyright 2023, Springer Nature. Transfer of millimetre‐sized 2D materials using tunable adhesive force tapes. Reproduced with permission.^[^ ^206^ ^]^ Copyright 2024, Springer Nature. Mass transfer of high‐density 2D FETs arrays. Reproduced with permission.^[^ ^207^ ^]^ Copyright 2025, Springer Nature. Down panels: M3D integration of 2D FETs for micro‐LED display. Reproduced with permission.^[^ ^191^ ^]^ Copyright 2021, Springer Nature. M3D integration of 2D FETs for ultimate edge computing. Reproduced with permission.^[^ ^192^ ^]^ Copyright 2023, Springer Nature. Low‐temperature M3D tier‐by‐tier integration of 2D FETs via vdW lamination. Reproduced with permission.^[^ ^208^ ^]^ Copyright 2024, Springer Nature. M3D integration of 2D FETs for static random‐access memory cell scaling. Reproduced with permission.^[^ ^205^ ^]^ Copyright 2025, Springer Nature. FeFETs for unltrahigh density 3D memory. Reproduced with permission.^[^ ^189^ ^]^ Copyright 2021, The American Association for the Advancement of Science. FeFETs for hardware implementation of neural networks, Reproduced with permission.^[^ ^190^ ^]^ Copyright 2023, Springer Nature. Low‐temperature nanosecond laser processing of FeFETs for M3D integration. Reproduced with permission.^[^ ^199^ ^]^ Copyright 2024, Wiley. Laser Processed M3D intergration of FeFET for neuromorphic computing. Reproduced with permission.^[^ ^201^ ^]^ Copyright 2025, Wiley.

This section provides an overview of recent advances in ferroelectric and 2D semiconductor platforms integrated with functional molecules for M3D integration. It highlights material breakthroughs, including synthesis and transfer techniques as well as device‐level demonstrations that underscore their potential in high‐density and multifunctional integration. **Figure** **5** portrays pivotal milestones in this evolution, charting the progression from material innovations to integrated device prototypes. In the following, we discuss the respective contributions of ferroelectrics, 2D semiconductors, and functional molecular switches to M3D integration, highlighting how each class of materials addresses distinct challenges and opportunities within this emerging paradigm.

FeFETs stand out in view of their compactness, energy efficiency, and compatibility with M3D integration schemes. A representative example is the realization of vertically stacked HZO‐based FeFETs, which enable Si‐free 3D integration with a large memory window of about 2.5 V and stable endurance over 10^8^ program/erase cycles. Device simulations further confirm the feasibility of ultrahigh‐density 3D ferroelectric memory integration, highlighting the potential of FeFETs for future 3D architectures.^[^ ^189^ ^]^ Building on this foundation, a 3D ferroelectric NAND array has been developed for area‐efficient neural‐network hardware, where each vertical layer functions as a hidden layer to perform parallel vector–matrix multiplications. This vertically stacked design achieves accurate color‐pattern classification and demonstrates a practical route toward energy‐efficient in‐memory computing using M3D ferroelectric platforms.^[^ ^190^ ^]^ Collectively, these advances position FeFETs as pivotal enablers of energy‐efficient, high‐density, and reconfigurable M3D computing systems. 2D semiconductors further complement M3D platform by offering dangling‐bond‐free surfaces that allow seamless integration with diverse substrates while eliminating lattice mismatch issues. Their atomic thickness also suppresses short‐channel effects and mitigates thermal cross‐talk, two critical bottlenecks in vertical stacking. Pioneering work has demonstrated 2D FETs are fully monolithic, low‐temperature, scalable and compatible with microelectronic processing, and can be used for advanced applications such as micro‐ light‐emitting diode display and ultimate edge computing.^[^ ^191^, ^192^ ^]^ As a proof‐of‐concept, Jariwala et al. proposed a 2D FeFET platform employing MoS_2_ channels and AlScN ferroelectrics, fully compatible with BEOL CMOS fabrication.^[^ ^25^ ^]^ Importantly, the maturing synthesis and transfer processes for both AlScN and MoS_2_—scaled up to 8‐inch^[^ ^193^ ^]^ and 12‐inch,^[^ ^194^ ^]^ respectively—demonstrate the feasible commercialization in the near future. To fully leverage their advantages in big data computing, further device miniaturization is essential. Recent advancements have shown that AlScN can be reliably sputter‐deposited down to 5 nm with retained ferroelectricity,^[^ ^195^ ^]^ setting the stage for integrating 2D semiconductors with ultra‐thin ferroelectrics, a critical step for achieving operational voltages compatible with cutting‐edge sub‐5 nm CMOS nodes. In parallel, progress in contact and channel engineering has significantly enhanced device‐level performance. The use of semi‐metallic bismuth as the contact electrode has enabled the formation of nearly‐ideal ohmic interfaces with monolayer transition metal dichalcogenides, drastically lowering the contact resistance.^[^ ^196^ ^]^ Furthermore, the realization of high‐performance p‐type 2D FETs through the integration of high work function metals or gate‐driven band modulation hydrodoping techniques has laid the groundwork for constructing CMOS‐compatible device platforms.^[^ ^197^ ^]^

Moreover, functional molecular switches also hold great promise in M3D integration, where they can serve as interfacial mediators bridging electronic, optical, and chemical functionalities across vertically stacked layers. When embedded at interlayer junctions, these molecules act as responsive dipolar linkers or charge‐transfer mediators, enabling signal coupling between neighboring tiers. In particular, photoresponsive molecular layers can be strategically positioned at optically accessible interfaces or integrated with transparent electrodes such as graphene or indium tin oxide (ITO), allowing efficient light penetration and activation even within multilayer stacks. Alternatively, guided optical coupling techniques—such as plasmonic or waveguide‐assisted delivery—can be utilized to direct light into buried molecular layers, enabling effective optical activation without compromising the compactness of the M3D stack. In terms of fabrication, their compatibility with low‐temperature solution processing further enhances their suitability for BEOL integration. Looking forward, site‐selective molecular assembly across tiers and the use of multifunctional molecules to couple memory, sensing, and computation within a unified vertical framework are anticipated. Such advances could give rise to a new generation of multifunctional M3D electronics that merge structural compactness with adaptive, multifunctional behavior. When combined with ferroelectric materials and 2D semiconductors, these molecular systems provide a third dimension of tunability, establishing a synergistic platform for M3D architectures.

While these material and device‐level advances highlight the promise of M3D integration, their practical implementation critically depends on compatibility BEOL processes. A central challenge for BEOL integration lies in reconciling the thermal budget, as the processing temperature of upper device layers stacked above CMOS circuits must remain below the critical threshold of 500 °C. Exceeding this limit risks dopant diffusion and performance degradation of underlaying layers.^[^ ^198^ ^]^ However, many ferroelectric materials, such as HfO_2_, typically require processing temperatures exceeding 600 °C, creating a fundamental conflict with BEOL integration. To overcome this challenge, a suite of low‐thermal‐budget processing strategies has been developed. Nanosecond pulsed laser annealing enables localized crystallization of HZO ferroelectrics by delivering sufficient thermal energy within a shallow penetration depth of 30–50 nm. Using a short laser wavelength of 355 nm and a pulse duration of 30 ns, the process achieves rapid cooling and precise heat confinement, inducing the required phase transformations with robust ferroelectricity without damaging underlying layers.^[^ ^199^ ^]^ In parallel, focused microwave annealing combines both electromagnetic and thermal energy to facilitate crystallization of HZO thin films at temperatures as low as 250 °C, yielding high‐performance M3D‐integrated FeFETs.^[^ ^200^ ^]^ Complementing these annealing‐based approaches, low‐temperature atomic layer deposition provides a direct route to deposit high‐quality hafnia‐based ferroelectric films at reduced processing temperatures. When combined with localized laser processing, it enables wafer‐scale fabrication of BEOL‐compatible FeFETs with record performance.^[^ ^201^ ^]^

Parallel efforts have targeted the low‐temperature synthesis of 2D semiconductors. A key breakthrough was achieved by Palacios et al., who developed a low‐thermal‐budget chemical‐vapor deposition approach to directly synthesize monolayer MoS_2_ on 200 mm silicon CMOS wafers at growth temperatures below 300 °C. This transfer‐free process not only preserves the electrical performance of underlying silicon circuits but also demonstrates wafer‐scale material uniformity with electron mobility of ≈35 cm^2^ V^−1^ s^−1^, thereby addressing synthesis scalability and uniformity, and establishing a BEOL‐compatible pathway for M3D integration.^[^ ^202^ ^]^ Building on this materials foundation, Chueh et al. demonstrated functional device integration by vertically stacking CVD‐grown MoS_2_ n‐type FETs with WSe_2_ p‐type FETs into M3D inverters on a 2.5 × 2.5 cm^2^ substrate. The entire fabrication was carried out below 250 °C, validated BEOL compatibility, and underscored the scalability of wafer‐level M3D logic circuits based on 2D semiconductors.^[^ ^203^ ^]^ Advancing beyond heterogeneous integration of distinct 2D semiconductors, Das et al. advanced to a single‐material platform by integrating complementary WSe_2_ FETs in separate vertical tiers interconnected through densely packed submicron vias. This architecture enabled transistor‐level partitioning and the demonstration of CMOS logic gates such as inverters, NAND, and NOR circuits, highlighting the applicability of 2D semiconductors for complex logic circuits.^[^ ^204^ ^]^ Extending this trajectory toward scaling, a 3‐tier M3D SRAM architecture based on monolayer MoS_2_ FETs was demonstrated, achieving up to 70% reduction in cell area, comparable to planar 250 nm nodes, thereby demonstrating the potential of vertical dimensional integration to sustain SRAM scaling at advanced nodes.^[^ ^205^ ^]^

In addition to low‐temperature processing routes, advanced transfer technologies have also emerged as powerful tools for realizing BEOL‐compatible integration of 2D semiconductors. Large‐area transfer of materials without compromising their crystalline quality enables their deterministic placement on pre‐fabricated circuits. Recent developments, such as UV‐controllable adhesive tapes with tunable adhesion strength, achieve near‐perfect transfer yield for 2D semiconductors while eliminating organic residues and solvent contamination.^[^ ^206^ ^]^ Likewise, microstructured polydimethylsiloxane‐based mass‐transfer printing allows wafer‐scale relocation and stacking of 2D arrays through capillary‐force‐assisted delamination, ensuring high yields and minimal mechanical damage.^[^ ^207^ ^]^ The technique enables damage‐free transfer of 2‐inch monolayer MoS_2_ films containing over one million arrays, with yields exceeding 99% and nearly pristine device performance. These solvent‐free, low‐stress transfer processes are inherently compatible with BEOL fabrication and complement direct low‐temperature growth, together forming an integrated toolkit for 3D stacking of functional layers. Benefiting from an advanced transfer technique, an M3D integrated system can be constructed via vdW lamination of prefabricated circuit tiers, with the processing temperature maintained at ≈120 °C. By sequentially repeating the vdW lamination process tier by tier, up to ten functional circuit layers can be vertically integrated, effectively overcoming the thermal budget limitations that previously constrained conventional 3D stacking approaches.^[^ ^208^ ^]^

Collectively, these advances establish a coherent roadmap in which low‐thermal‐budget ferroelectric processing, BEOL‐compatible 2D semiconductor synthesis, and molecular functionalization converge to enable M3D integration. By reconciling the stringent thermal constraints of BEOL fabrication with the demand for multifunctional, high‐performance operation, these approaches transform M3D from a conceptual framework into a technologically credible pathway. Beyond merely sustaining Moore's law through density scaling, M3D architectures enriched by ferroelectrics, 2D materials, and molecular systems point toward a new paradigm of vertically integrated electronics that simultaneously achieve energy efficiency, functional diversification, and adaptive reconfigurability. In doing so, they address the long‐standing challenges of silicon‐based scaling—power consumption, interconnect latency, and thermal management—while laying the groundwork for compute‐in‐memory accelerators, edge AI platforms, and ultradense memory systems. Thus, M3D integration emerges not only as an engineering solution to extend scaling but also as a transformative materials platform to redefine the trajectory of information technologies in the post‐Moore era.

## 6 Cutting‐Edge Material Science for Broader Applications

The evolution of material science is driving groundbreaking innovations that address critical challenges in next‐generation electronic devices. Among these advancements, we highlight four representative examples—spanning energy‐efficient computing, wearable electronics, nanoscale data storage, and thermal management—each illustrating the transformative potential of advanced materials in reshaping modern technology.

### 6.1 Polymers with Giant Electrostrictive Properties for 2D Electrostrictive FETs (2D EFETs)

**Figure 6:**
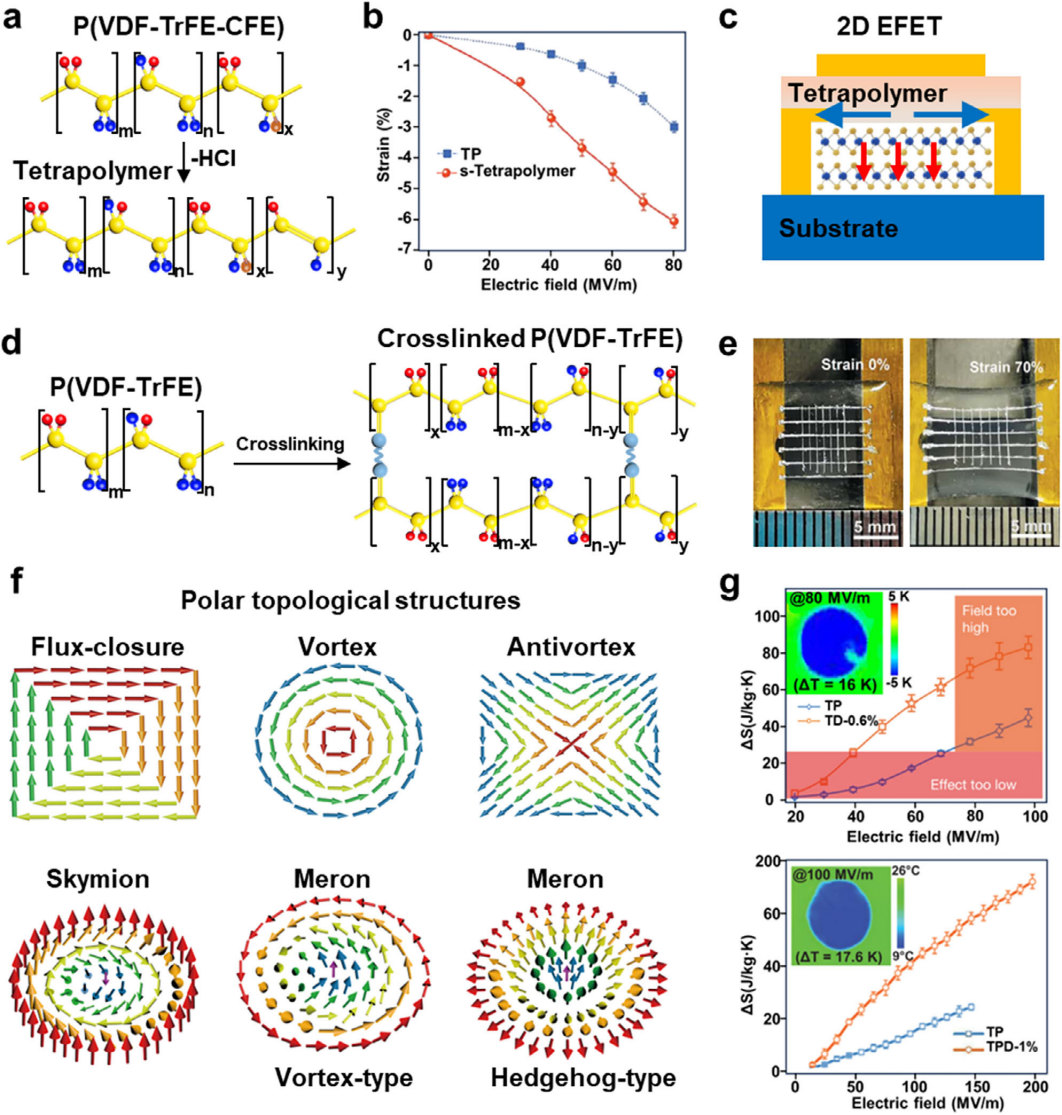
Cutting‐edge material science for broader applications. a) Synthesis of tertrapolymer with giant electrostrictive properties, and b) its thickness strain at 1‐Hz unipolar field versus electric field. Reproduced with permission.^[^ ^209^ ^]^ Copyright 2022, The American Association for the Advancement of Science. c) Schematic illustration of a 2D EFET. d) Synthesis of an elastic polymer by slight cross‐linking, and e) corresponding elastic device under 0 and 70% strain. Reproduced with permission.^[^ ^214^ ^]^ Copyright 2023, The American Association for the Advancement of Science. f) Schematics of typical topological structures in ferroelectric materials. Reproduced with permission.^[^ ^230^ ^]^ Copyright 2021, Wiley. g) Giant electrocaloric effect in tertrapolymer (up), and interface‐augmented polymer (down). Reproduced with permission.^[^ ^233^, ^234^ ^]^ Copyright 2021, 2023, The American Association for the Advancement of Science.

The development of ferroelectric tertrapolymers with giant electrostrictive properties marks a significant milestone in the advancement of 2D EFETs. These tetrapolymers were obtained by introducing a small amount of fluorinated alkyne monomers (<2 mol.%) into the relaxor ferroelectric terpolymer poly(vinylidene fluoridetrifluoroethylene‐chlorofluoroethylene) P(VDF‐TrFE‐CFE), resulting in markedly enhanced polarization change with strong electromechanical coupling while suppressing non‐contributing polarization processes (**Figure** **6**a).^[^ ^209^ ^]^ With an electromechanical coupling factor of ≈88% and a piezoelectric coefficient exceeding 1000 pm V^−1^, the tetrapolymer can efficiently generate mechanical stress under low bias, making it well‐suited as the gate dielectric in 2D EFETs (Figure 6b).

The 2D EFET operates on the principle of voltage‐induced strain transduction. In this configuration, the electrostrictive tetrapolymer serves as the stress‐generating gate oxide, while 2D semiconductors with exceptional strain sensitivity act as the channel (Figure 6c). Upon electrical biasing, the polymer undergoes longitudinal expansion, generating out‐of‐plane stress on the 2D channel. This stress dynamically reduces the interlayer distance of the semiconductor, thereby narrowing its bandgap. As a result, the device transitions reversibly from a wide‐bandgap OFF state to a nearly gapless ON state.^[^ ^210^, ^211^, ^212^ ^]^ As a consequence of this efficient strain transduction, internal voltage amplification occurs within the channel, enabling sub‐60 mV dec^−1^ SS, a key benchmark for low‐power, high‐efficiency electronics.

While this approach offers exciting opportunities to transcend conventional semiconductor limits, its practical implementation faces significant interfacial challenges. Weak vdW interactions at the ferroelectric‐2D semiconductor interface impede efficient strain transfer, constraining the full exploitation of the tetrapolymer's giant electrostrictive response. Addressing this bottleneck will require advanced interfacial engineering strategies, such as chemical functionalization, adhesion‐layer design, or epitaxial matching, to strengthen mechanical coupling. Beyond electrostrictive polymers, alternative device architectures employing piezoelectric gate stacks or hybrid heterostructures may offer complementary pathways to achieve robust strain transfer and ultra‐steep SS.^[^ ^213^ ^]^ Continued progress in this area is essential to fully harness the potential of 2D EFETs for next‐generation adaptive and low‐power electronics.

### 6.2 Elastic and Biodegradable Ferroelectrics for Wearable Electronics

Wearable electronics are becoming increasingly essential in modern healthcare, yet their reliance on batteries remains a significant limitation. Ferroelectric materials, with their intrinsic piezoelectricity, offer a sustainable solution by harvesting mechanical energy from human motion into electrical energy. In particular, fluoropolymers, owing to their inherent molecular flexibility and excellent processability, naturally align with the requirements of flexible and conformable electronics. While for practical use in wearable systems, however, ferroelectrics must combine mechanical elasticity with stable polarization responses under repeated strain.

Recent advances have demonstrated that polymer‐based ferroelectrics can be elastified while preserving their functionality. For example, slight crosslinking has successfully infused ferroelectric polymers like P(VDF‐TrFE) with elasticity, allowing them to maintain stable ferroelectric responses under strains of up to 70% (Figure 6d,e).^[^ ^214^ ^]^ High dielectric constants are also essential for low‐voltage operation and power consumption. Advances in the elastification of relaxor ferroelectric materials have shown promise. For instance, peroxide crosslinked P(VDF‐CTFE‐DB)^[^ ^215^ ^]^ and P(VDF‐TrFE‐CFE)^[^ ^216^ ^]^ achieve dielectric constants around 19.4 and 54.2 at 100 Hz, respectively, while preserving ferroelectric stability up to 80% strain. Such properties enable stretchable, energy‐harvesting platforms capable of powering low‐power electronics.

Biodegradability represents another pivotal feature, particularly for implantable devices. In this regard, molecular ferroelectrics, inherently biodegradable, have emerged as promising candidates.^[^ ^217^ ^]^ While some of their properties still lag behind conventional ferroelectrics, some exhibit polarization levels comparable to ceramic counterparts. To improve device‐grade quality, volume‐confinement methods have been used to grow ultrasmooth single‐crystal ferroelectric thin films at nanometer to micrometer thickness with sub‐centimeter scale.^[^ ^218^ ^]^ Moreover, hybrid perovskite‐based inverters integrating memory and logic functions have demonstrated the feasibility of molecular ferroelectrics for logic‐in‐memory devices.^[^ ^219^ ^]^ Further exploration of elastification techniques will be key to adapting these materials for dynamic biological environments.^[^ ^220^ ^]^

In light of growing concerns over fluorine‐based “forever chemicals”, a new class of fluorine‐free ferroelectric polymers has recently been developed, featuring a polyoxypropylene main chain with disulfonyl alkyl side chains connected by a C3 spacer (−SO_2_CH_2_CHRCH_2_SO_2_−, R = H or CH_3_).^[^ ^221^ ^]^ Strong dipole–dipole interactions between neighboring disulfonyl groups induce ferroelectric ordering in the condensed state, where R = H yields classical ferroelectricity, while R = CH_3_ leads to relaxor ferroelectricity. Notably, the relaxor polymer exhibits electroactuation and electrocaloric performance comparable to state‐of‐the‐art PVDF‐based tetrapolymers, while completely eliminating fluorinated components. This advancement represents a major step toward environmentally sustainable, biocompatible, and high‐performance ferroelectrics suitable for flexible and implantable electronic applications.

Collectively, elastic and biodegradable, and fluorine‐free ferroelectrics bridge critical gaps in next‐generation wearable systems, enabling real‐time health monitoring, body‐conforming implants, and smart devices tailored for the Internet of Medical Things (IoMT). When integrated with in‐memory computing architectures, these systems can further evolve toward intelligent sensing and on‐device data processing. Such integration allows physiological signals to be directly converted, stored, and analyzed within the same platform, paving the way for energy‐efficient AI‐enabled medical diagnostics.

### 6.3 Polar Topological Structures for (Sub)Nanoelectronics

Ferroelectric materials offer exceptional promise for advanced nanodevices due to their fine polarization control, especially in data storage and processing. However, as storage densities escalate to gigabits per square inch and individual storage dimensions shrink to nanoscale, size effects begin to critically impact data storage, retrieval, and integrity, underscoring the urgent need for innovative storage solutions.

First‐principle calculations have predicted a range of polar topological structures in ferroelectric materials,^[^ ^222^ ^]^ featuring nanometer‐scale spatially varying polarization orientations without critical thickness limitations. Diverse polar topologies, such as flux‐closure,^[^ ^223^, ^224^ ^]^ vortices/antivortices,^[^ ^225^, ^226^ ^]^ skyrmion,^[^ ^227^, ^228^ ^]^ and merons,^[^ ^229^ ^]^ have been observed in perovskites, polymers, and 2D ferroelectric materials (Figure 6f).^[^ ^230^ ^]^ These nanoscale polar structures effectively minimize cross‐talk between adjacent bits and are robust against external perturbations. Theoretically, they enable storage density exceeding 60 Tbit/in^2^, surpassing current devices using ferroelectric domains as storage units. Recent experimental advances have demonstrated rewritable polar textures on silicon platforms, switchable via electric fields, establishing a pathway toward logic‐in‐memory hardware.^[^ ^228^ ^]^ Notably, resonant subterahertz excitation has been employed to initiate collective dynamics of polar vortices, enabling data processing on picosecond timescales.^[^ ^225^ ^]^ This breakthrough opens exciting prospects for terahertz optoelectronics, ultrafast data exchange, and intra‐chip communication in emerging computer circuits.

Despite these promising advances, research into polar topologies remains in its infancy, leaving ample room for further investigation. To gain deeper insights into these topological polar structures, atomic‐resolution techniques such as transmission electron microscopy (TEM) are essential. However, conventional TEM techniques primarily provide 2D atomic projections, insufficient for understanding the 3D dipole structures of individual polar configurations. With advancements of cutting‐edge TEM technology, recent progress in atomic electron tomography allows for the observation of atomic‐level 3D arrangement of polar topology in nanoparticles.^[^ ^231^ ^]^ Moreover, innovations in electron microscopy hardware and technologies, such as electron ptychography, have improved spatial resolution to as fine as 0.39 Å, providing new possibilities for more precise elucidation of polar topological structures in ferroelectrics.^[^ ^232^ ^]^

Looking forward, controlled array formation of topological domains remains a grand challenge. Achieving stable electrode integration, deterministic switching, and scalable fabrication protocols for addressing individual polar textures are essential milestones. Progress in these directions will not only deepen our fundamental understanding of ferroelectric topologies but also pave the way for topologically encoded memory and signal‐processing hardware in beyond‐CMOS nanoelectronics.

### 6.4 Polymers with Giant Electrocaloric Effect for Chip Cooling

As chip integration density and computational performance continue to advance, thermal dissipation has become a major bottleneck. Excessive heat accumulation not only degrades device reliability but can also lead to irreversible failure. In this context, the electrocaloric effect in ferroelectric materials, which induces reversible temperature and entropy changes through the application of an electric field, has emerged as a promising solid‐state cooling strategy for on‐chip thermal regulation. A key challenge, however, is that significant electrocaloric performance typically requires high electric fields, which risks dielectric breakdown and material fatigue. Recent breakthroughs in ferroelectric polymers have addressed this limitation. Qian et al. reported tetrapolymers derived from P(VDF‐TrFE‐CFE), in which a fraction of polar chlorofluoroethylene groups was chemically converted into covalent double bonds.^[^ ^233^ ^]^ This modification increases the number of polar entities and expands the polar‐nonpolar interfacial area, resulting in an enhanced entropy change of 16 K under 80 MV m^−1^ (Figure 6g). Building on this concept, an advanced strategy involves introducing organic crystal dimethylhexynediol as a 3D sacrificial master to assemble multiconformation polar interfaces within the terpolymer matrix. The resulting polymer achieved an unprecedented temperature change of 17.6 K under 100 MV m^−1^ (Figure 6g).^[^ ^234^ ^]^ In addition, the strong electrostriction effect exhibited by these high‐entropy polymers has enabled the design of self‐oscillating polymeric refrigerators with high energy efficiency, capable of regulating the chip temperature with various sizes and thermal loads.^[^ ^235^ ^]^

Despite these advances, the reliance on bulky external high‐voltage power supplies for device operation and testing hampers the miniaturization and portability of electrocaloric cooling modules. A feasible pathway involves integrating compact, high‐voltage power supplies directly into the device structure, paired with custom control circuit logic and microcontrollers to regulate the high‐voltage signal output. These advances would enable portable and scalable thermal management, facilitating the continued miniaturization of electronic devices while reducing energy consumption and environmental impact.

Collectively, the four material innovations discussed in this section—giant electrostrictive polymers, elastic and biodegradable ferroelectrics, polar topological structures, and colossal electrocaloric polymers—demonstrate a unified trend toward functional convergence, structural miniaturization, and system‐level integration. These advanced materials not only break traditional performance boundaries but also expand the functionality of electronic systems across diverse domains, including neuromorphic computing, wearable healthcare, ultradense memory, and self‐regulating thermal platforms. Looking ahead, future efforts should emphasize the co‐design of materials and device architectures to enable seamless integration of these materials into heterogeneous, monolithic, and even bio‐integrated systems. In parallel, scalable fabrication, reliability under harsh conditions, and full CMOS compatibility need to be addressed to realize commercial translation. By bridging molecular design, interfacial engineering, and system‐level innovation, these advanced materials are poised to redefine the frontiers of electronics, ushering in a new era of intelligent, adaptive, and sustainable device technologies.

## 7 Conclusion and Outlook

Hybrid systems comprising ferroelectrics, 2D semiconductors, and molecular switches have emerged as transformative platforms addressing post‐Moore demands for simultaneous high integration density and multifunctional performance. This Review has systematically surveyed 2D FeFET applications spanning non‐volatile memories, neuromorphic computing, reconfigurable logic circuits, NC‐FETs, and multifunctional FeFETs, while identifying critical bottlenecks and mitigation strategies. Subsequently, from a materials viewpoint, it discusses advancements in nanoscale downscaling of key ferroelectrics—including perovskite oxides, hafnia‐based ferroelectrics, fluoropolymers, vdW ferroelectrics and molecular ferroelectrics—while offering a critical assessment of nanoscale integration strategies. It reveals how the incorporation of functional molecular switches enables to expansion the multifunctionality of 2D FeFETs, that combined with their advances in M3D integration, open new pathways for emerging high‐performance ferroelectrics with significant yet underexplored device‐level potential.

Despite notable progress, research on the hybrid systems remains in its embryonal stages, with several key challenges yet to be addressed. At the material level, wafer‐scale, high‐quality, atomically thin films have been achieved only in a limited set of early‐studied materials, such as doped HfO_2_ and select 2D semiconductors like transition metal dichalcogenides. To enable broader adoption, there is a pressing need for low‐cost, scalable, and high‐throughput synthesis and transfer techniques that are compatible with industrial requirements. At the device level, most reported 2D FeFETs remain at the proof‐of‐concept stage, typically demonstrated as single or small‐scale devices. Large‐scale integration is hindered not only by the lack of robust platforms for evaluating system‐level performance metrics—such as stability, endurance, and fault tolerance—but also by the absence of scalable fabrication technologies. Therefore, the development of CMOS‐compatible, low‐temperature processes is critical to enable the seamless transition from laboratory‐scale prototypes to fully integrated electronic systems. At the fundamental level, maintaining and enhancing ferroelectric polarization in the nanoscale regime remains challenging, as depolarization fields intensify with decreasing film thickness. Moreover, the origin and switching dynamics of extrinsically induced ferroelectricity, such as that arising from interlayer sliding, are still not well understood. Finally, the coupling mechanisms between ferroelectric layers and 2D semiconducting channels, which govern key device characteristics such as hysteresis, switching speed, and retention, remain to be fully elucidated.

Looking ahead, unlocking the full potential of hybrid systems will require close integration between materials innovation, device engineering, and physical modelling. Key research directions include achieving atomic‐level control over ferroelectric domains, improving interfacial stability and dipolar coupling, and developing system‐level architectures that can exploit multifunctional responses under low‐power operation. In particular, advances in M3D integration, scalable molecular functionalization, and in situ characterization techniques will be essential for bridging the gap between proof‐of‐concept devices and manufacturable platforms. As the field continues to evolve, these hybrid systems are poised to play a central role in the development of intelligent, adaptive, and energy‐efficient electronics that transcend the limits of conventional semiconductor technologies.

## Conflict of Interest

The authors declare no conflict of interest.
